# D-dimer levels and characteristics of lymphocyte subsets, cytokine profiles in peripheral blood of patients with severe COVID-19: A systematic review and meta-analysis

**DOI:** 10.3389/fmed.2022.988666

**Published:** 2022-10-05

**Authors:** Haiyue Zhang, Huajun Wu, Dongli Pan, Weifeng Shen

**Affiliations:** Department of Clinical Laboratory, The First Hospital of Jiaxing and The Affiliated Hospital of Jiaxing University, Jiaxing, China

**Keywords:** COVID-19, D-dimer, lymphocyte subsets, cytokine, meta-analysis

## Abstract

**Purpose:**

A series of complications caused by severe COVID-19 can significantly affect short-term results. Therefore, early diagnosis is essential for critically COVID-19 patients. we aimed to investigate the correlation among D-dimer levels, lymphocyte subsets, cytokines, and disease severity in COVID-19 patients.

**Methods:**

Systematic review and meta- analysis of PubMed, Scopus, Web of Science, Cochrane Central Register of Controlled Trials, Embase, clinical trials, and China National Knowledge Infrastructure (CNKI) until 1 August 2022. We considered case-control, and cohort studies that compared laboratory parameters between patients with severe or non-serious diseases or between survivors and non-survivors. Pooled data was assessed by use of a random-effects model and used *I*^2^ to test heterogeneity. We assessed the risk of bias using the Newcastle- Ottawa Scale.

**Results:**

Of the 5,561 identified studies, 32 were eligible and included in our analysis (*N* = 3,337 participants). Random-effect results indicated that patients with COVID-19 in severe group had higher levels for D-dimer (WMD = 1.217 mg/L, 95%CI=[0.788, 1.646], *P* < 0.001), neutrophil-to-lymphocyte ratio (NLR) (WMD = 6.939, 95%CI = [4.581, 9.297], *P* < 0.001), IL-2 (WMD = 0.371 pg/ml, 95%CI = [−0.190, 0.932], *P* = 0.004), IL-4 (WMD = 0.139 pg/ml, 95%CI = [0.060, 0.219], *P* = 0.717), IL-6 (WMD = 44.251 pg/ml, 95%CI = [27.010, 61.493], *P* < 0.001), IL-10 (WMD = 3.718 pg/ml, 95%CI = [2.648, 4.788], *P* < 0.001) as well as lower levels of lymphocytes (WMD = −0.468( × 10^9^/L), 95%CI = [−0.543, −0.394], *P* < 0.001), T cells (WMD = −446.746(/μL), 95%CI = [−619.607, −273.885], *P* < 0.001), B cells (WMD = −60.616(/μL), 95%CI = [−96.452, −24.780], *P* < 0.001), NK cells (WMD = −68.297(/μL), 95%CI = [−90.600, −45.994], *P* < 0.001), CD3+T cells (WMD = −487.870(/μL), 95%CI = [−627.248, −348.492], *P* < 0.001), CD4+T cells (WMD = −290.134(/μL), 95%CI = [−370.834, −209.435], *P* < 0.001), CD8+T cells (WMD = −188.781(/μL), 95%CI = [−227.806, −149.757], *P* < 0.001).

**Conclusions:**

There is a correlation among higher levels of D-dimer, cytokines, lower levels of lymphocyte subsets, and disease severity in COVID-19 patients. These effective biomarkers may help clinicians to evaluate the severity and prognosis of COVID-19. This study is registered with PROSPERO, number CRD42020196659.

**Systematic review registration:**

https://www.crd.york.ac.uk/PROSPERO/display_record.php?RecordID=196659; PROSPERO registration number: CRD42020196659.

## Introduction

The current 2019 Novel Coronavirus (2019-nCoV) infection reportedly originated in Wuhan, Hubei Province, China in December 2019. After being declared a pandemic on March 11, 2020, it has affected more than 200 countries / Areas of 40 million people (1). Countries around the world have entered a state of emergency, and everyone can feel the impact on health, business, and other aspects of daily life. According to reports, the most common initial symptoms of COVID-19 are fever, cough, fatigue, anorexia, and diarrhea (2). Shortness of breath occurs on average 5 to 8 days after the initial symptoms appear; its appearance indicates that the condition has deteriorated (3). Although most patients with COVID-19 pneumonia have a good prognosis, some patients develop to acute respiratory distress syndrome (ARDS), coagulation disorders, or multiple organ failure, with a mortality rate between 4 and 15% (4, 5). More timely and effective early intervention for severe patients is a priority. And early diagnosis is the best approach to achieve this aim. Therefore, we believe that comprehensive monitoring of the severity of COVID-19 and effective early intervention are basic measures to reduce the mortality rate. Some studies have reported several abnormal hematological parameters in patients with COVID-19, including lymphopenia, neutrophilia, elevated levels of D-dimer, and fibrinogen (6–9).

Besides, inflammatory cytokine levels are obvious laboratory abnormalities observed during infection with COVID-19 (10, 11). However, the clinical significance of these biomarkers has not been fully clarified. This meta-analysis aims to reveal the characteristics of laboratory test results in patients with COVID-19 through the included articles, especially the changes in severely ill patients, to define which parameters can distinguish those who are at higher risk of severe and non-serious diseases.

## Methods

### Search strategy and selection criteria

This meta-analysis is reported following with the Preferred Reporting Items for Systematic Reviews and Meta-Analyses (PRISMA) Statement and has been registered at the International Prospective Register of Systematic Reviews (numberCRD42020196659) (12).

We search PubMed/MEDLINE, Scopus, Web of Science, Cochrane Central Register of Controlled Trials, Embase, clinical trials, China National Knowledge Infrastructure (CNKI) and Google Scholar databases for articles published until 1 August 2022, using the keywords. “coronavirus,” “2019-nCoV,” “COVID-19,” “SARS-CoV-2,” “2019 novel coronavirus disease,” “coronavirus disease 2019,” “laboratory,” “clinical characteristics.” In addition, the WHO publication database, lancet, New England Journal of Medicine, JAMA, BMJ were screened to find potentially relevant publications. To ensure the comprehensiveness and accuracy of the research, we also consulted the references of the attached literature.

Study designs eligible for inclusion were cross sectional, case-control, and cohort studies. To be eligible, the studies must clearly indicate that the patients have been diagnosed as COVID-19 and were positive for SARS-CoV-2RNA. Studies were excluded if patients were asymptomatic carriers and did not fulfill the inclusion criteria. Studies were considered eligible for inclusion in this meta-analysis if they compared laboratory parameters between patients with severe or non-serious diseases or between survivors and non-survivors. Since the pre-prints have not been peer-reviewed, we did not include these papers in our analysis to avoid the spread of any potential misinformation. The following studies were excluded: duplicate publications, reviews, editorials, single case reports, small case series (< 10 cases), studies did not include the biomarkers required for the meta-analysis or missing research data. Two authors independently screened the title and abstract based on these selection criteria, discussed the differences with another author and subsequently resolved through negotiation.

### Data extraction and quality assessment

Two authors (HYZ, HJW) independently extracted the data, differences were resolved in a discussion, or, if a consensus could not be reached, the third author resolved (WFS). We extracted the following variables: first author; publication year; study design; country; the number of participants in severe and non-severe disease groups; levels of laboratory indexes (d-dimer levels, lymphocyte subsets, cytokines) in different groups. If further information is needed, we will email the corresponding author, if there is no response, we will exclude the study. Stratified data or interquartile range (IQR) were converted to mean (±SD) using mathematical formulas for meta-analysis (13, 14). We planned to use the Cochrane Risk of Bias tool to assess the risk of bias in randomized trials, but our search did not find any eligible randomized trials. We used the Newcastle-Ottawa scale to rate the risk of bias in the non-randomized study. There are 8 criteria for NOS, with scores ranging from 0 (high-risk deviation) to 9 (low-risk deviation). Studies with a NOS score> 7 were considered high- quality.

### Statistical analysis

We used Stata (version 12.0) for all statistical analysis. We used the I^2^ statistic and Cochran's *Q*-test to assess statistical heterogeneity. We believe that an I^2^ statistics of 0–25% shows a low heterogeneity, the medium heterogeneity is 26–75%, and the high heterogeneity is 76–100%, the heterogeneity *p*-value is < 0.05. If there is heterogeneity, the random effect model was used, otherwise, the fixed-effect model was used. Weighted mean difference (WMD) with 95% confidence intervals (95% CI) was calculated for D-dimer, lymphocyte subsets, and cytokines. We studied the influence of a single study on the overall risk estimate and eliminated each study in each round to test the robustness of the main results. If a meta- analysis included more than ten studies, publication bias was assessed by Begg and Egger test. We defined significant publication bias as a *p*-value < 0.05.The trim-and-fill computation was used to estimate the effect of publication bias on the interpretation of the results.

## Results

### Literature search and studies characteristics

**Figure 1:**
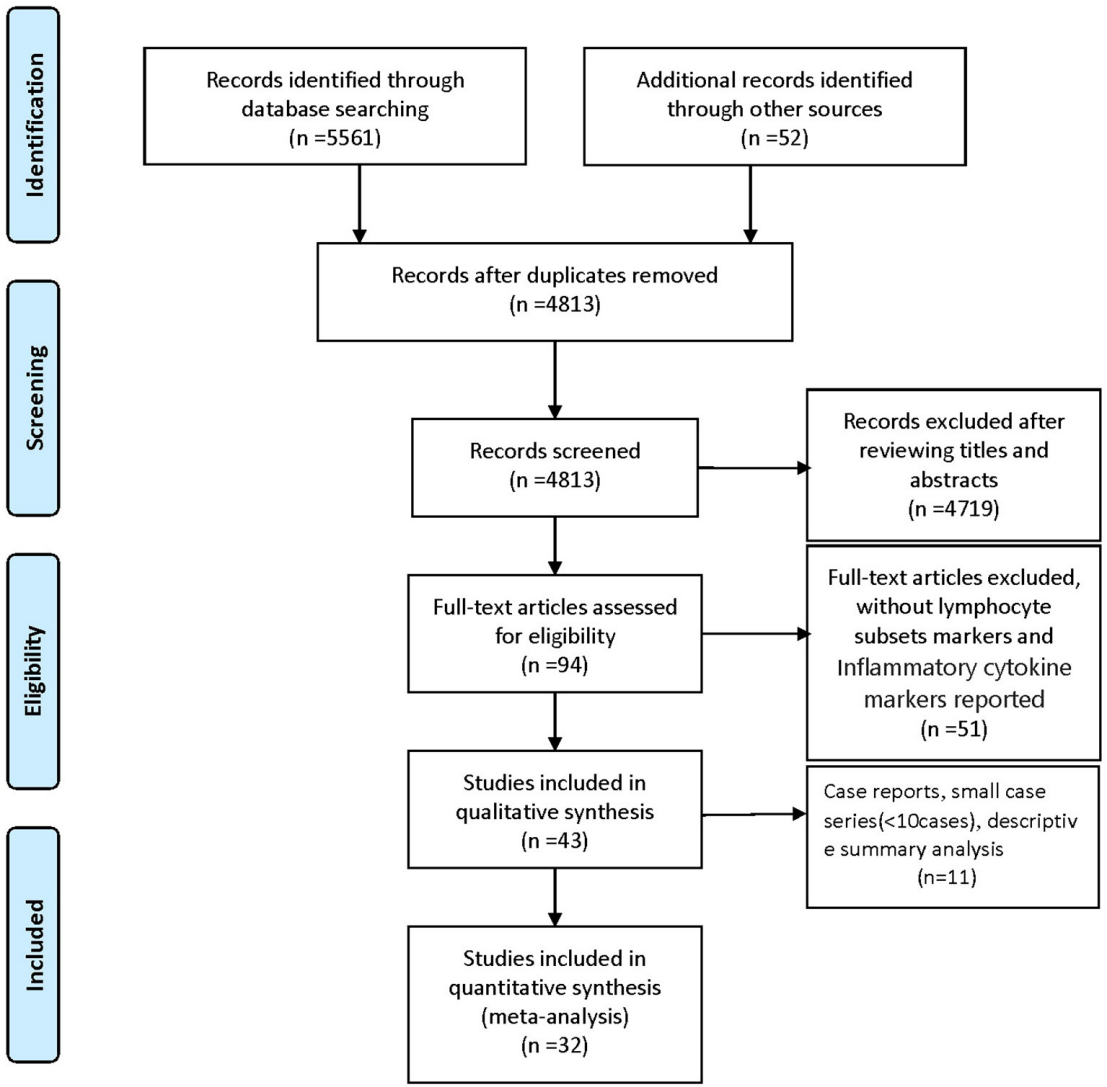
Literature search and filtering of studies.

We identified 5561 studies, of which 32 (data from 3337 participants) were included in our analysis (10, 11, 15–44) (Figure 1). The basic characteristics of the articles included in the study are shown in Table 1. Five of the 32 trials were published in 2022, four trials in 2021, and 23 trials were published in 2020. Two studies were from Italy, two studies were from Turkey, one study was from Germany, one study was from the United States, one study was from Spain, 1 study was from India, one study was from the Republic of North Macedonia, and the rest were from China. Twenty-eight studies were grouped by severity and non-severity, and four studies were grouped by survivors and non-survivors of COVID-19. Among these articles, 21 articles described the D-dimer levels of COVID-19 patients, 28 articles described the lymphocyte subsets levels of COVID-19 patients, 17 articles described the cytokine levels of COVID-19 patients. All the included studies were rated high quality according to the NOS scores and details are shown in Table 2.

**Table 1:** Characteristics of enrolled studies in the meta-analysis.

**Author (first)**	**Year**	**Country**	**Groups**	**Case**	**Age**	**Sex (men, %)**	**Case definition (who)**	**Biomarkers**	**Quality**
Ufuk O. Idiz	2022	Turkey	Severe and non-severe	31	60.6 ± 14.5	19(61.2)	Confirm	D-dimer, Lymphocytes, neutrophil-to-lymphocyte ratio, IL-6, IL-10	9
Ibrahim Koc	2022	Turkey	Severe and non-severe	226	55.9 ± 1.5	136(60.2)	Confirm	D-dimer, Lymphocytes, neutrophil-to-lymphocyte ratio	8
Zendelovska	2022	Republic of North Macedonia	Severe and non-severe	50	55 ± 11.4	unknown	Confirm	IL-6, CD8+	7
Hongye Wang	2022	China	Severe and non-severe	37	53.5(48.5–66)	16(43.2)	Confirm	D-dimer, Lymphocytes, B cells, NK cells	9
Luca Masotti	2022	Italy	Survivors and non-survivors	223	69.4 ± 13.3	121(54.3)	Confirm	D-dimer, Lymphocytes, neutrophil-to-lymphocyte ratio, IL-6	9
Jing Zhang	2021	China	Survivors and non-survivors	208	64(53–72)	129(62)	Confirm	D-dimer, Lymphocytes, IL-2, IL-6, IL-10, T cells, B cells, NK cells, CD4+, CD8+	9
Yueting Tang	2021	China	Severe and non-severe	71	60(47–68)	27(38)	Confirm	Lymphocyte, d-dimer	9
Mukta Pujan	2021	India	Severe and non-severe	100	46–65 years age group(68)	64(64)	Confirm	D-dimer, IL-6, neutrophil-to-lymphocyte ratio	8
Mercedes Garc'ia-Gasalla	2021	Spain	Severe and non-severe	120	59(49.5–71)	64(53.3)	Confirm	D-dimer, IL-6, IL-10, neutrophil-to-lymphocyte ratio, CD3+, CD4+, CD8+, CD4+/CD8+, NK cells	9
Xun Li	2020	China	Survivors and non-survivors	25	73(55–100)	10(40)	Confirm	Lymphocyte, d-dimer	7
Tao Chen	2020	China	Survivors and non-survivors	274	62.0(44.0–70.0)	171(62.4)	Confirm	lymphocyte, d-dimer, IL-6, IL-10	9
Xiaotian Dong	2020	China	Severe and non-severe	18	58.4 ± 17.2	11(61.1)	Confirm	D-dimer, IL-10, IL-2, IL-4, IL-6, TNF-α, Lymphocytes, T cells, B cells, CD4+/CD8+, NK cells	9
Ruyuan He	2020	China	Severe and non-severe	204	49(34–62)	79(38.7)	Confirm	Lymphocyte count, D-dimer, CD3+, CD4+, CD8+, CD4+/CD8+, NK cells, IL-2, IL-10	7
Ying Sun	2020	USA/ China	Severe and non-severe	63	47(3–85)	37(58.7)	Confirm	Lymphocytes, D-dimer, IL-6, T ly, B ly, CD4+, CD8+, NK cells, CD4+/CD8+	9
Jing Liu	2020	Germany/ China	Severe and non-severe	40	48.7 ± 13.9	15(37.5)	Confirm	Lymphocyte, d-dimer	8
Yang Liu	2020	China	Severe and non-severe	76	45(18–78)	49(64.5)	Confirm	Lymphocyte, D-dimer, IL-6, IL-8, CD8+	7
Jianhong Fu	2020	China	Severe and non-severe	75	46.6 ± 14	45(60)	Confirm	Lymphocyte, d-dimer, neutrophil-to-lymphocyte ratio	7
Hongbo Shi	2020	China	Severe and non-severe	54	62.67 ± 10	22(40.7)	Confirm	Lymphocyte	8
Ai-Ping Yang	2020	China	Severe and non-severe	93	46.4 ± 17.6	56(60.2)	Confirm	neutrophil-to-lymphocyte ratio, CD3+, CD4+/CD8+, NK cells, D-dimer	7
Suxin Wan	2020	China	Severe and non-severe	123	52.17 ± 14.34	66(53.7)	Confirm	Lymphocyte, CD4+, CD8+, CD4+T/CD8+, B cells, NK cells, IL-4, IL-6, IL-10	8
Rui Liu	2020	China	Severe and non-severe	154	64 ± 14	84(54.5)	Confirm	Lymphocyte, CD3+, CD4+, CD8+, CD4+/CD8+, NK cells	7
Zhe Zhu	2020	China	Severe and non-severe	127	15.26	45(35.4)	Confirm	Lymphocyte, Neutrophil-to-lymphocyte ratio, D-dimer, IL-2, IL-4, IL-6, IL-10	7
Da-wei Sun	2020	China	Severe and non-severe	57	61.7 ± 12.8	29(50.9)	Confirm	Lymphocytes, T cells, B cells, NK cells	7
Chuan Qin	2020	China	Severe and non-severe	452	58(47–67)	235(52.0)	Confirm	lymphocytes, T cells, B cells, NK cells, Neutrophil-to-lymphocyte ratio, IL-6, IL-10	7
Yang Yang	2020	China	Severe and non-severe	50	62(22–78)	29(58)	Confirm	Lymphocyte, CD4+, CD8+	9
Miriana d'Alessandro BS	2020	Italy	Severe and non-severe	22	63(59-68)	15(68.2)	Confirm	CD3+, CD4+, CD8+, NK cells, CD4+/CD8+	7
Ying Chi	2020	China	Severe and non-severe	78	43.31 ± 2.42	42(53.8)	Confirm	IL-2, IL-4, IL-6, IL-10	9
Bo XU	2020	China	Severe and non-severe	187	62(48.5–71)	103(55.1)	Confirm	CD3+, CD4+, CD8+, CD4+/CD8+, B cells, NK cells, IL-6, IL-10, Lymphocytes, D-dimer	8
Ruchong Chen	2020	China	Severe and non-severe	548	56.0 ± 14.5	313(57.1)	Confirm	Lymphocyte, Neutrophil-to-lymphocyte ratio, CD3+, CD4+, CD8+, CD4+/CD8+, IL-6, D-dimer	9
Haizhou Wang	2020	China	Severe and non-severe	95	55 ± 16	51(53.7)	Confirm	Lymphocyte, CD3+, CD4+/CD8+, NK cells	7
Guang Chen	2020	China	Severe and non-severe	21	56(50–65)	17(81.0)	Confirm	Lymphocyte, D-dimer, CD4+, CD8+, NK cells	9
Ming Ni	2020	China	Severe and non-severe	27	60(33–83)	14(51.9)	Confirm	Lymphocytes, CD4+, CD8+, T cells, B cells, NK cells	7

**Table 2:** Methodological quality of enrolled studies based on Newcastle-Ottawa Scale (NOS).

**Study**	**Is the definition adequate?**	**Representativeness of the cases**	**Selection of controls**	**Definition of controls**	**Comparability of cases and controls on the basis of the design or analysis**	**Ascertainment of exposure**	**Same method of asertalnment for cases and controls**	**Non-response rate**	**Total scores**
Idiz et al. (15)	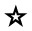	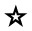	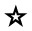	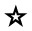	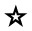 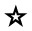	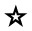	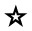	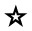	9
Koc et al. (16)	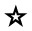	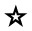	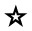	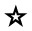	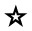	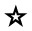	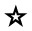	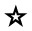	8
Zendelovska et al. (17)	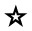	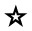	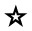	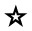	-	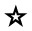	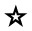	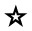	7
Wang et al. (18)	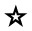	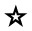	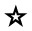	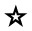	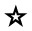 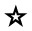	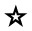	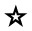	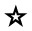	9
Masotti et al. (19)	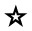	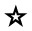	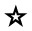	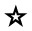	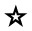 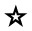	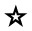	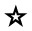	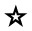	9
Zhang et al. (20)	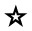	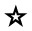	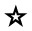	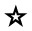	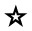 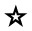	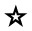	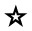	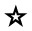	9
Tang et al. (21)	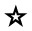	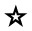	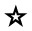	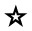	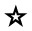 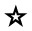	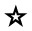	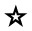	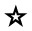	9
Pujan et al. (22)	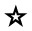	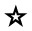	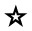	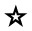	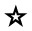	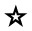	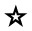	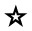	8
Garc'ia-Gasalla et al. (23)	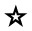	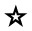	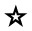	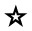	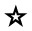 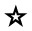	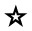	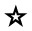	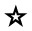	9
Li et al. (24)	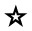	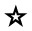	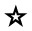	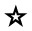	-	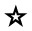	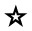	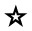	7
Chen et al. (25)	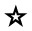	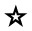	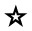	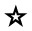	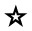 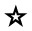	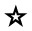	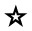	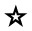	9
Dong et al. (26)	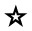	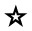	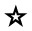	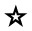	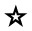 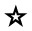	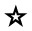	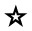	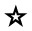	9
He et al. (27)	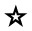	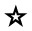	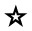	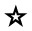	-	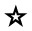	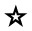	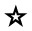	7
Sun et al. (28)	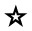	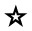	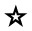	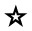	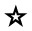 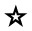	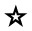	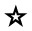	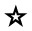	9
Liu et al. (29)	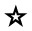	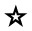	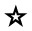	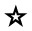	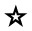	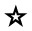	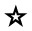	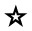	8
Liu et al. (30)	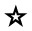	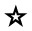	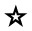	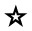	-	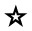	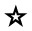	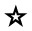	7
Fu et al. (31)	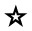	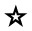	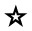	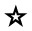	-	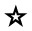	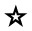	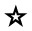	7
Shi et al. (32)	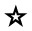	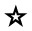	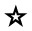	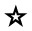	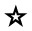	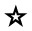	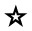	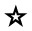	8
Yang et al. (33)	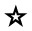	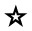	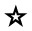	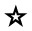	-	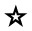	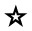	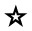	7
Wan et al. (34)	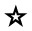	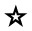	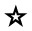	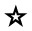	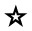	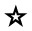	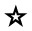	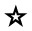	8
Liu et al. (35)	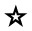	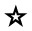	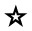	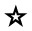	-	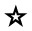	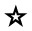	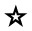	7
Zhu et al. (36)	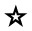	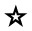	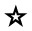	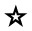	-	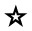	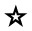	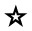	7
Sun et al. (37)	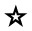	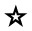	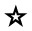	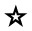	-	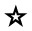	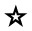	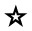	7
Qin et al. (38)	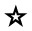	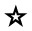	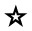	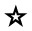	-	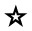	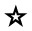	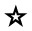	7
Yang et al. (10)	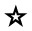	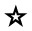	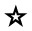	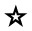	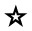 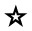	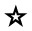	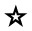	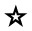	9
d'Alessandro et al. (39)	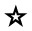	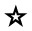	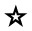	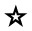	-	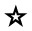	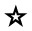	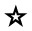	7
Chi et al. (40)	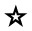	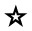	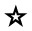	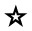	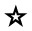 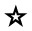	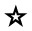	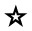	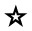	9
Xu et al. (41)	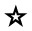	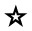	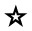	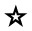	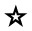	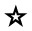	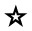	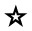	8
Chen et al. (38)	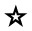	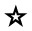	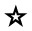	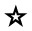	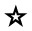 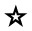	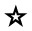	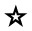	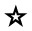	9
Wang et al. (18)	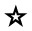	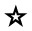	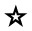	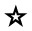	-	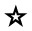	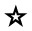	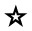	7
Chen et al. (11)	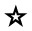	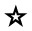	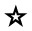	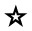	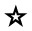 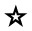	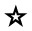	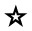	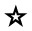	9
Ni et al. (42)	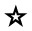	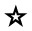	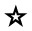	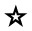	-	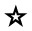	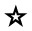	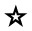	7

### Association of d-dimer levels in the peripheral blood with the severity of COVID-19

**Figure 2:**
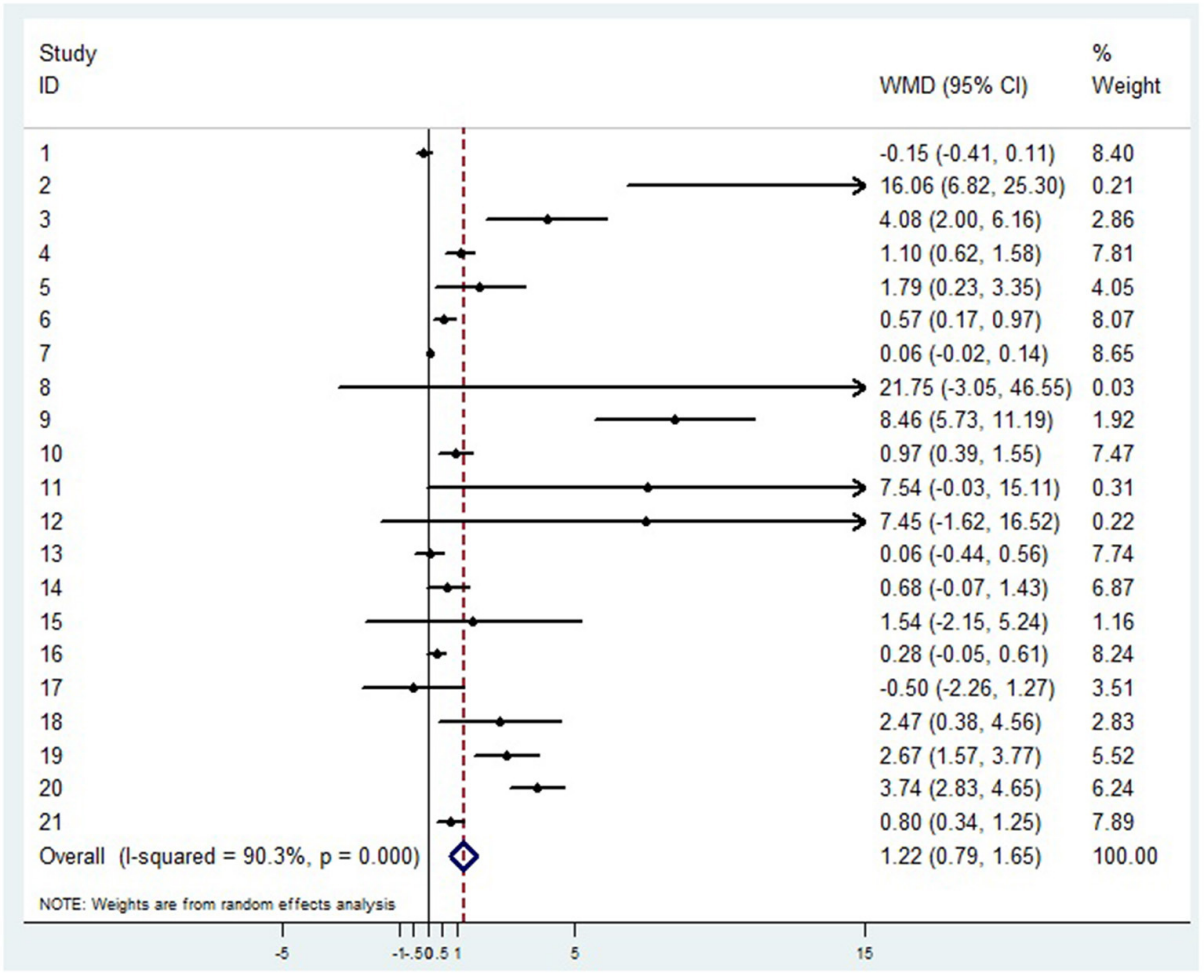
Forest plot between non-severe and severe groups for levels of D-dimer.

Twenty-one articles showed that compared with patients in non-severe group, patients in severe group had higher levels of D-dimer (WMD = 1.217 mg/L, 95%CI = [0.788, 1.646], *P* < 0.001) (Figure 2). A sensitivity analysis was carried out by excluding one study at a time and reanalyzing the entire data set. We found that the results were not influenced by excluding any one specific study (Supplementary material 1A). A funnel plot based on the d-dimer levels of patients showed *p*-values of 0.001 in Egger's test (Supplementary material 1B). There was publication bias in the studies included in the meta-analysis. After filling one trial, the revised result was still consistent using random model (WMD = 0.306 mg/L, 95%CI = [−0. 167, 0.778], *P* = 0.205) or fix model (WMD = 0.127 mg/L, 95%CI = [0.058, 0.195], *P* < 0.001) (Supplementary material 1C). Besides, using standard mean difference (SMD) for the meta-analysis still did not change the conclusion (Table 3).

**Table 3:** The results of the meta-analysis based on standard mean difference (SMD).

**Biomarkers**	**Studies**	**Participants**	**Heterogeneity**		**Model**	**SMD**	**95%CI**	***P***
			***I* ^2^**	***P***				
D-dimer	21	2397	90.3%	< 0.001	Random	0.869	[0.596, 1.141]	< 0.001
Lymphocytes	24	2368	75.5%	< 0.001	Random	−1.054	[−1.343, −0.764]	< 0.001
T cells	7	948	76.5%	< 0.001	Random	−1.104	[−1.396, −0.813]	0.253
B cells	10	1172	87.5%	< 0.001	Random	−0.854	[−1.515, −0.193]	< 0.001
NK cells	17	1881	85%	< 0.001	Random	−0.805	[−1.080, −0.529]	< 0.001
CD3+T cells	8	1423	88.4%	< 0.001	Random	−1.316	[−1.712, −0.921]	< 0.001
CD4+T cells	13	1820	93.1%	< 0.001	Random	−1.664	[−2.287, −1.041]	< 0.001
CD8+T cells	15	1853	93.6%	< 0.001	Random	−1.977	[−2.564, −1.390]	< 0.001
Neutrophil-to-lymphocyte ratio(NLR)	11	1995	88.3%	< 0.001	Random	1.177	[0.832, 1.523]	< 0.001
IL-2	4	427	60.6%	0.055	Random	0.521	[0.020, 1.023]	0.042
IL-4	4	346	81.2%	0.001	Random	0.592	[−0.224, 1.409]	0.155
IL-6	17	2904	98.9%	< 0.001	Random	1.400	[0.977, 1.822]	< 0.001
IL-10	11	1635	93%	< 0.001	Random	1.628	[1.032, 2.223]	< 0.001

### The decrease of lymphocyte subsets in severe patients with COVID-19

**Figure 3:**
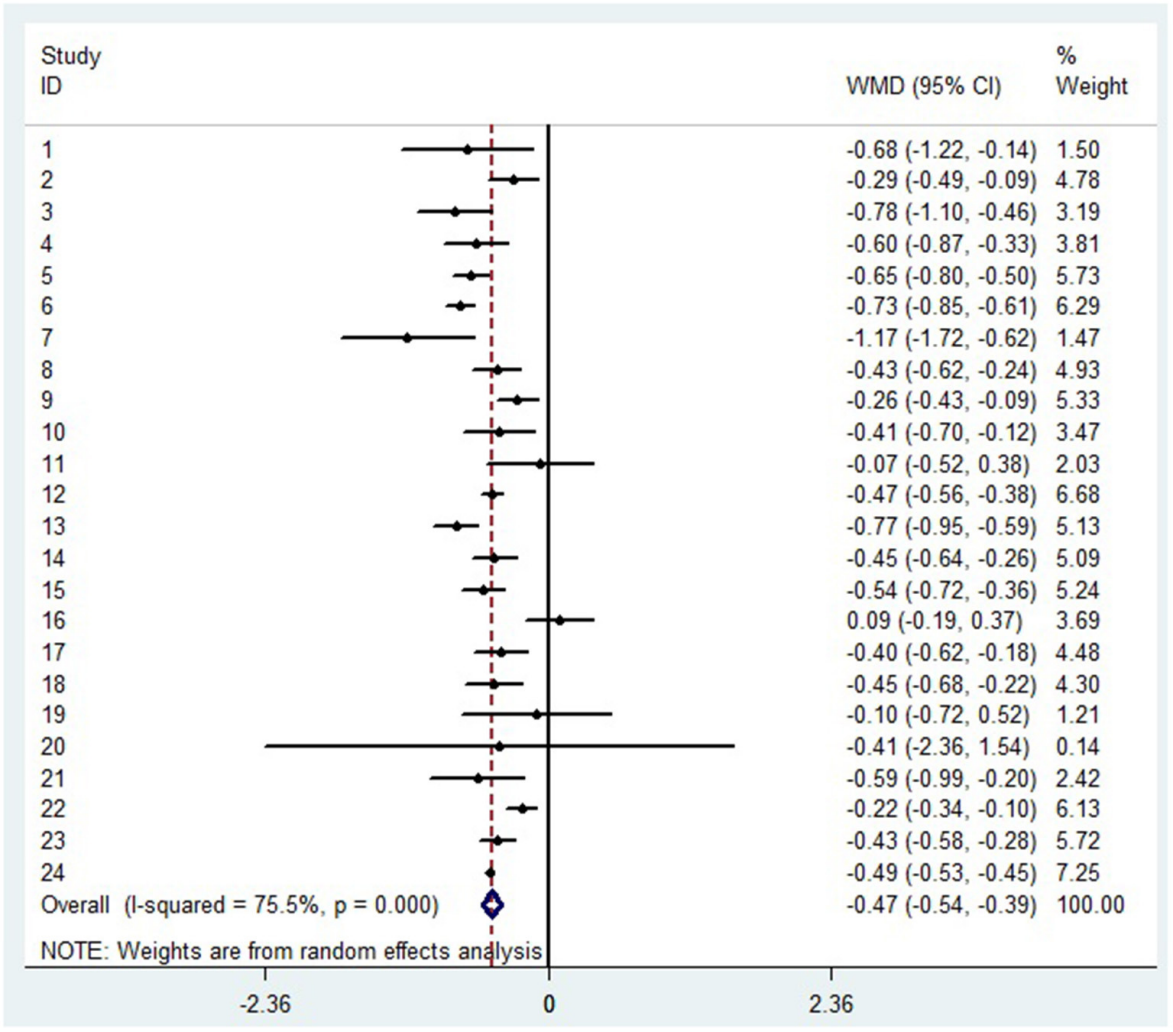
Forest plot between non-severe and severe groups for levels of lymphocytes.

**Figure 4:**
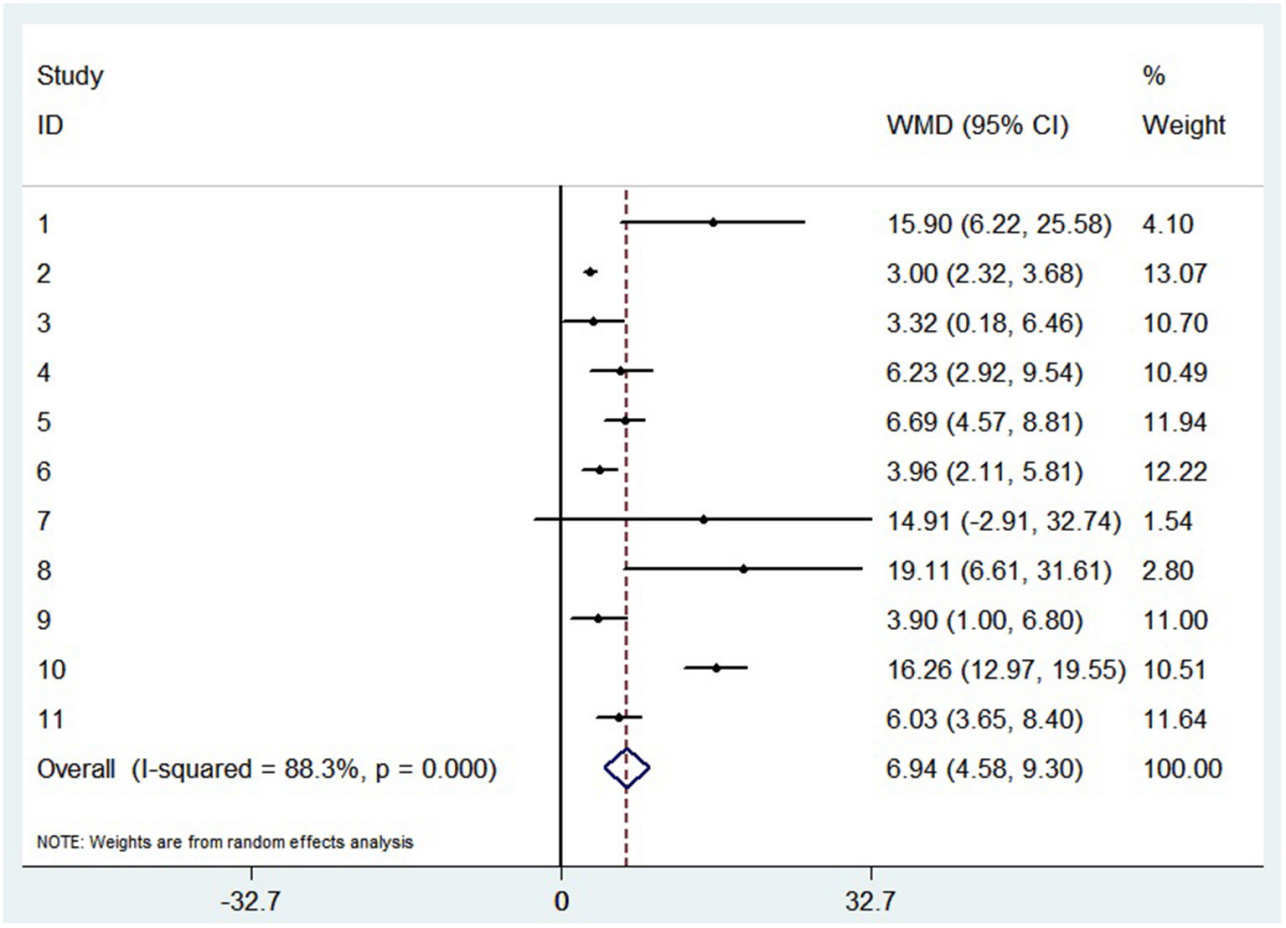
Forest plot between non-severe and severe groups for levels of neutrophil-to-lymphocyte ratio (NLR).

A random-effects model was used to analyze the correlation between the level of patient lymphocyte subsets and the severity of COVID-19. Compared with the non-severe group, patients in the severe group had lower levels for lymphocytes (WMD = −0.468( × 10^9^/L), 95%CI = [−0.543, −0.394], *P* < 0.001) (Figure 3), T cells (WMD = −446.746 (/μL), 95%CI = [−619.607, −273.885], *P* < 0.001), B cells(WMD = −60.616(/μL), 95%CI = [−96.452, −24.780], *P* < 0.001), NK cells (WMD = −68.297(/μL), 95%CI = [−90.600, −45.994], *P* < 0.001), CD3+T cells (WMD = −487.870(/μL), 95%CI = [−627.248, −348.492], *P* < 0.001), CD4+T cells (WMD = −290.134(/μL), 95%CI = [−370.834, −209.435], *P* < 0.001), CD8+T cells (WMD = −188.781(/μL), 95%CI = [−227.806, −149.757], *P* < 0.001) (Supplementary materials 2A–F). Furthermore, the neutrophil-to-lymphocyte ratio (NLR) (WMD = 6.939, 95%CI = [4.581, 9.297], *P* < 0.001) (Figure 4) of severe patients was higher than that of non-severe patients. Sensitivity analyses indicated that the results were robustness by removing any one specific study in all the lymphocyte subsets and NLR between non-severe and severe groups (Supplementary materials 2G–N). No significant publication bias was detected in most of the studies except for NK cells (*p* = 0.034) and neutrophil-to-lymphocyte ratio (NLR) (*p* = 0.016) (Supplementary materials 2O–T). When applying the trim-and-fill method, there were no any trials trimmed and filled in NK cells and neutrophil-to-lymphocyte ratio(NLR) (Supplementary materials 2U,V). Besides, using standard mean difference (SMD) for the meta-analysis still did not change the conclusion (Table 3).

### The increase of cytokines in peripheral blood of patients with COVID-19

**Figure 5:**
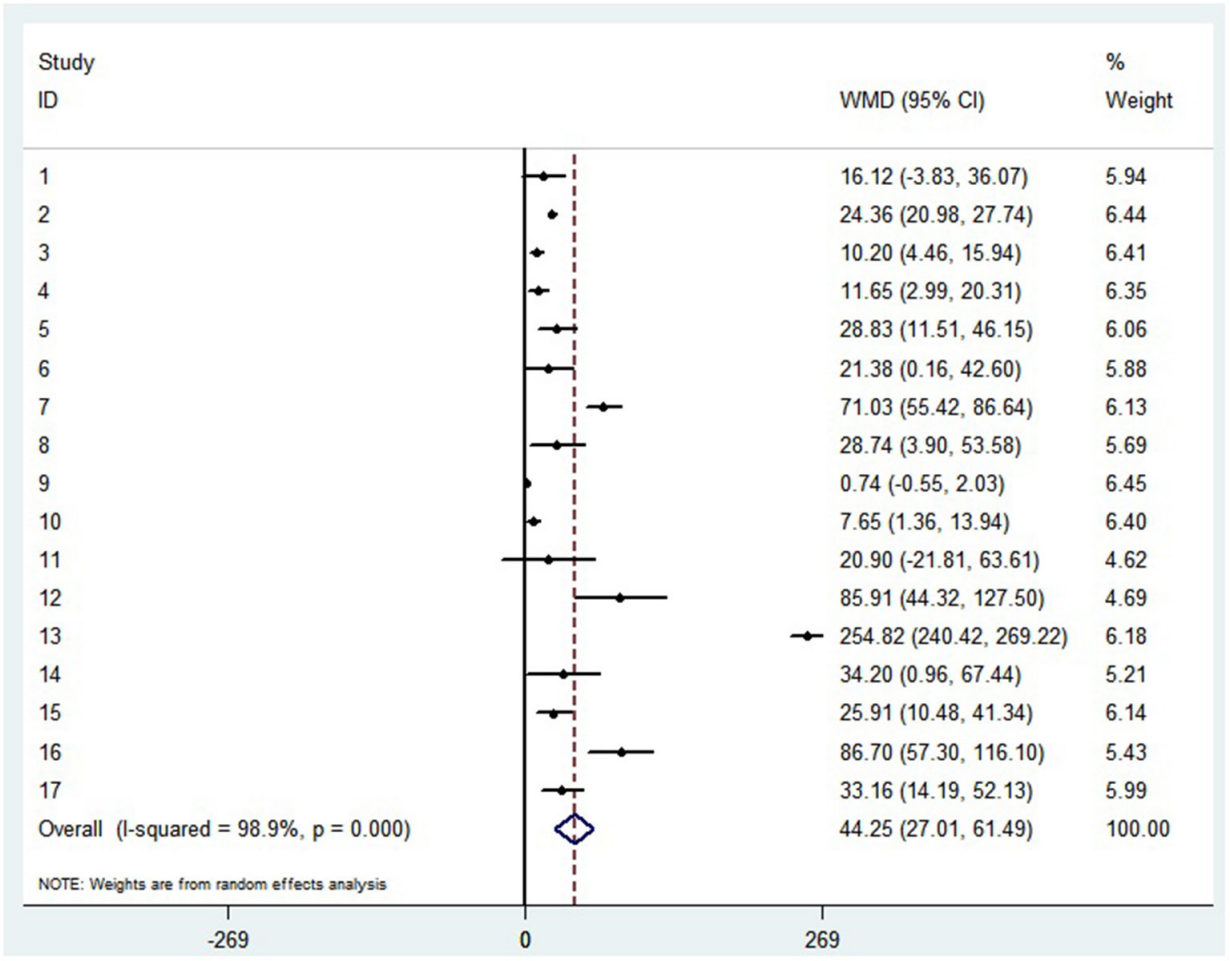
Forest plot between non-severe and severe groups for levels of IL-6.

The random-effect results demonstrated that compared with patients in the non-severe group, patients in the severe group had higher levels for interleukin (IL)-2 (WMD = 0.371 pg/ml, 95%CI = [−0.190,0.932], *P* = 0.004), IL-4 (WMD = 0.139 pg/ml, 95%CI = [0.060, 0.219], *P* = 0.717) (Supplementary materials 3A,B), IL-6 (WMD = 44.251 pg/ml, 95%CI = [27.010, 61.493], *P* < 0.001) (Figure 5), IL-10 (WMD = 3.718 pg/ml, 95%CI = [2.648, 4.788], *P* < 0.001) (Supplementary material 3C). Sensitivity analysis by removing one study in each turn, the result indicated that the main result was robustness (Supplementary materials 3D–G). A funnel plot based on the IL-6 levels of patients showed *p*-values of 0.031 in Egger's test (Supplementary material 3H). No significant publication bias was detected in IL-10 (Supplementary material 4I). When applying the trim-and-fill method, there were no any trials trimmed and filled in IL-6 (Supplementary material 3J). Besides, using standard mean difference (SMD) for the meta-analysis still did not change the conclusion (Table 3).

## Discussion

This meta-analysis showed that higher levels of d-dimer, cytokines, NLR, and lower levels of lymphocyte subsets were associated with the severity of COVID-19 infection.

Several studies have shown that the inflammatory response plays a key role in COVID-19, excessive elevation of inflammatory cytokines can cause the so-called “cytokine storm,” and the inflammatory cytokine storm increases the severity of COVID-19 (45, 46). The fifth edition of “Diagnosis and Treatment of COVID-19” recommends monitoring the cytokine levels to improve treatment effectiveness and reduce mortality (47). Siddiqi et al. (48) found that inflammatory cytokines and biomarkers are significantly elevated during the systemic inflammation stage of COVID-19 and excessive hyper inflammation may lead to cardiopulmonary collapse and multi-organ failure. Several studies have shown that the blood coagulation function is significantly activated during severe COVID-19 infection, which may be related to the sustained inflammation response caused by the release of cytokines induced by virus invasion. Recent lung pathological anatomy evidence showed that pulmonary small vessel occlusion and micro thrombosis in critically ill patients with COVID-19 (7). The most significant coagulation marker is the significant and dynamic increase of D-dimer levels. However, the etiology of elevated serum D-dimer levels is multifactorial. The significant inflammatory response in patients with severe COVID-19 may increase the possibility of thromboembolic disease, which may explain the increase in serum d-dimer levels. COVID-19-related coagulopathy needs special attention and treatment. In the absence of contraindications, it is recommended to use a preventive dose of anticoagulant for all COVID-19 patients.

Many studies have reported lymphopenia in patients with COVID-19, because SARS-CoV-2 particles may destroy the cytoplasmic components of lymphocytes and cause apoptosis (8). For the SARS virus, He et al. (9) suspected that lymphocytes and their subsets are essential to eliminating virus-infected cells, while for COVID-19 Henry B hypothesized that survival may depend on the ability to replenish lymphocytes and their subsets killed by the virus (31). Therefore, lymphocyte count may be used as a clinical predictor of severity and prognosis. Previous studies have shown that the severity of SARS pathological damage is related to the extensive infiltration of neutrophils in the lung and the increase in the number of peripheral blood neutrophils (49). Therefore, the increase in neutrophil count may indicate the intensity of the inflammatory responses in patients with COVID-19. Besides, the degree of lymphopenia also indicates course and severity of the COVID-19. Thus, NLR may have a potential value in monitoring the condition of severe COVID-19 patients.

In terms of immune biomarkers, elevated IL-6, IL-10, along with elevated C-reactive protein (CRP) and procalcitonin (PCT), indicating that patients with severe diseases have systemic inflammatory response syndrome (SIRS). Additionally, elevated IL-10 may be related to compensatory anti-inflammatory response (CARS), which may be the cause of secondary infection in the severe group and non-survival group (50). Therefore, we suggest that these parameters can be used to monitor the prognosis of COVID-19 patients during hospitalization.

However, some limitations in our meta-analysis should be mentioned. Firstly, the number of cases is small. Secondly, Most of the included studies were from China. As more data from other countries becomes available, further investigation is needed. Lastly, the language of studies was limited to English, which may result in potential language bias.

In conclusion, our analysis showed that higher levels of D-dimer, NLR, cytokines (IL-2, IL-4, IL-6, IL-10) and lower levels of lymphocyte subsets in severe patients, which are of great significance for predicting disease changes. For hospitalized patients, we recommend that clinicians closely monitor D-dimer levels, lymphocyte subsets, and cytokines as markers for potential progression to severe disease. Finally, these parameters should continue to be re-evaluated in the future, because more data can be obtained in future large cohort studies.

## Data availability statement

The original contributions presented in the study are included in the article/Supplementary material, further inquiries can be directed to the corresponding author/s.

## Author contributions

HZ and WS were responsible for study design. HZ and DP were involved in data collection. HZ and HW analyzed the data. HZ wrote the manuscript. HZ, HW, DP, and WS revised the manuscript. All authors contributed to the article and approved the submitted version.

## Funding

This work was funded by the Zhejiang Medicine and Health under Grant 2022RC077, the Venus Talent Training of the First Hospital of Jiaxing of Zhejiang Province of China under Grant 2020-QMX-25.

## Conflict of interest

The authors declare that the research was conducted in the absence of any commercial or financial relationships that could be construed as a potential conflict of interest.

## Publisher's note

All claims expressed in this article are solely those of the authors and do not necessarily represent those of their affiliated organizations, or those of the publisher, the editors and the reviewers. Any product that may be evaluated in this article, or claim that may be made by its manufacturer, is not guaranteed or endorsed by the publisher.
