# Supplementary material for: D-dimer levels and characteristics of lymphocyte subsets, cytokine profiles in peripheral blood of patients with severe COVID-19: A systematic review and meta-analysis
Source: Front Med (Lausanne). 2022 Oct 5;9:988666. doi: 10.3389/fmed.2022.988666 (PMC9579342; doi:10.3389/fmed.2022.988666)

Supplement 1

(A)Sensitivity analyses between nonsevere and severe groups for levels of D-dimer.

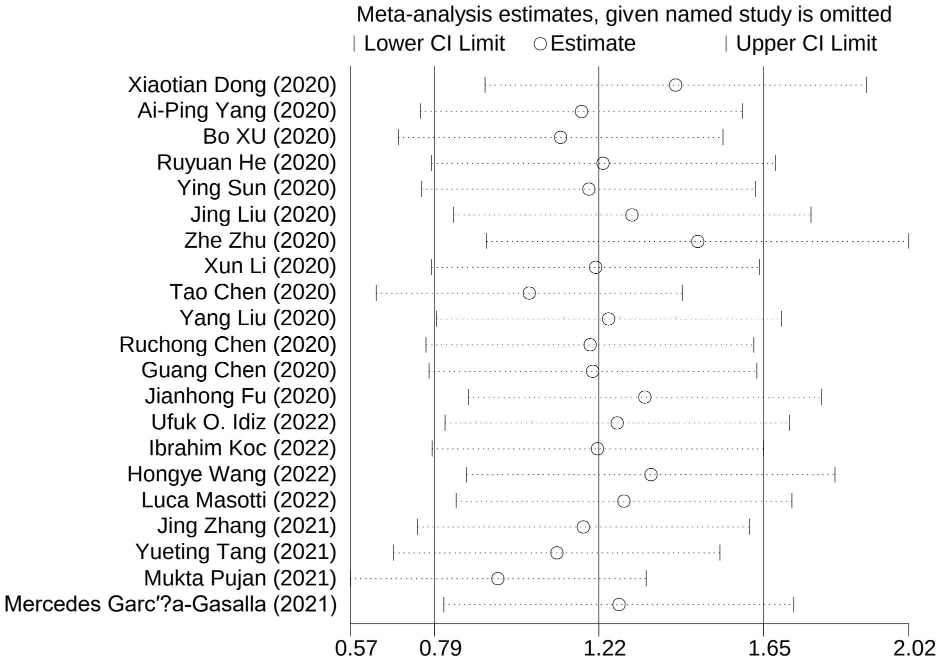

(B)Publication bias by Egger' test between nonsevere and severe groups in D-dimer.

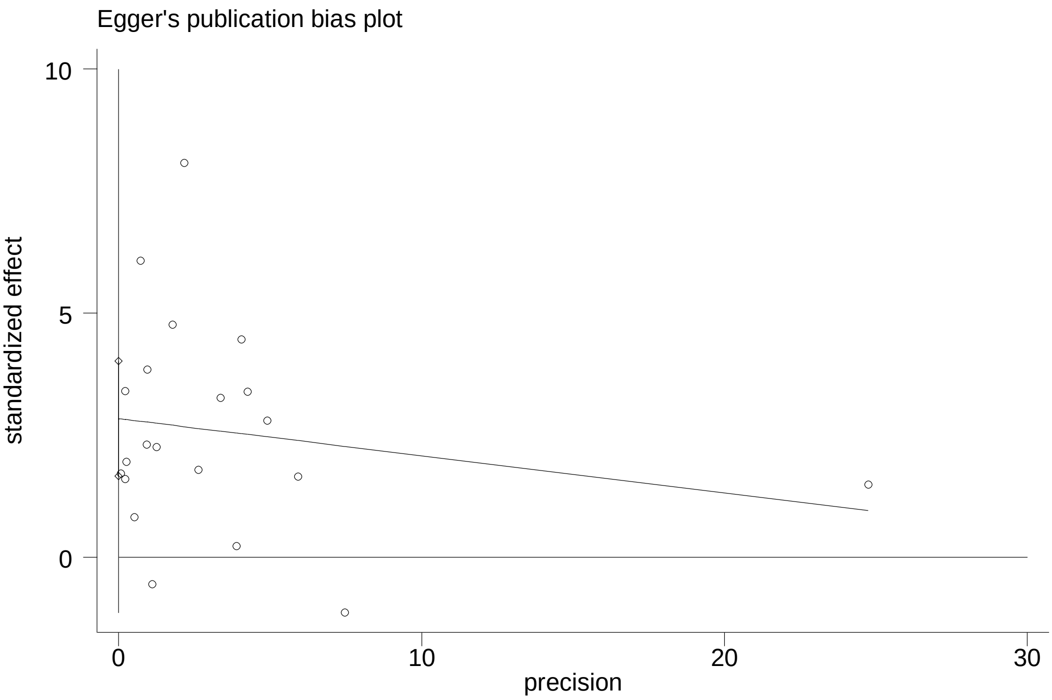

Supplement 1

(C)Funnel plot by trim-and-fill method between nonsevere and severe groups in D-dimer.

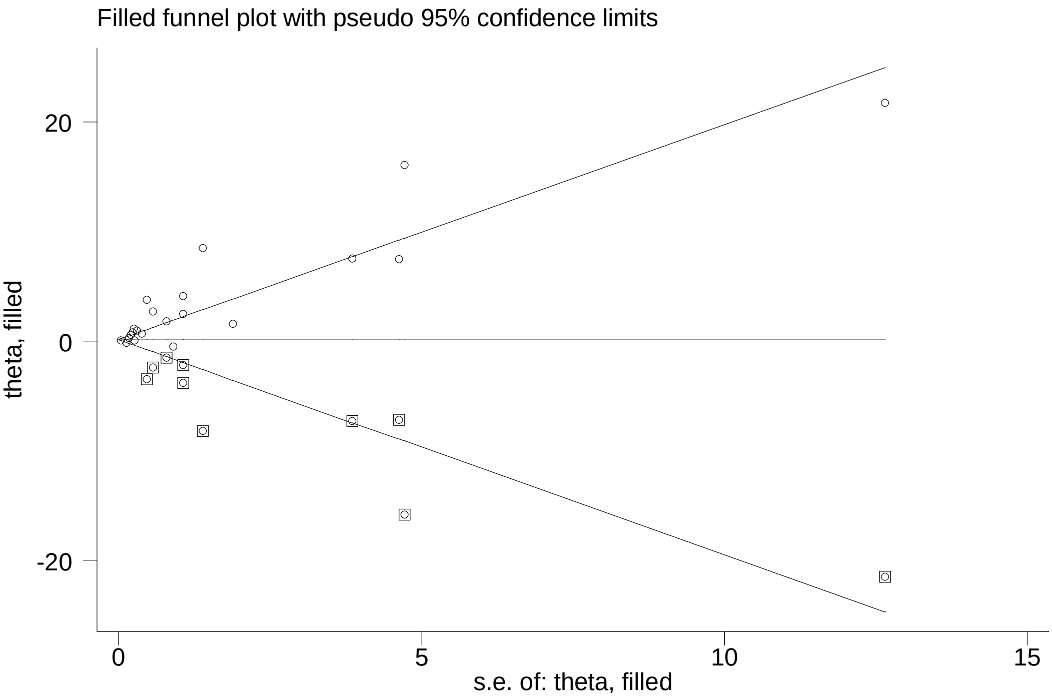

Supplement: Supplementary file 1 [file Data_Sheet_1.pdf]
