# Supplementary material for: D-dimer levels and characteristics of lymphocyte subsets, cytokine profiles in peripheral blood of patients with severe COVID-19: A systematic review and meta-analysis
Source: Front Med (Lausanne). 2022 Oct 5;9:988666. doi: 10.3389/fmed.2022.988666 (PMC9579342; doi:10.3389/fmed.2022.988666)

Supplement 2

(A) Forest plot between nonsevere and severe groups for levels of T cells.

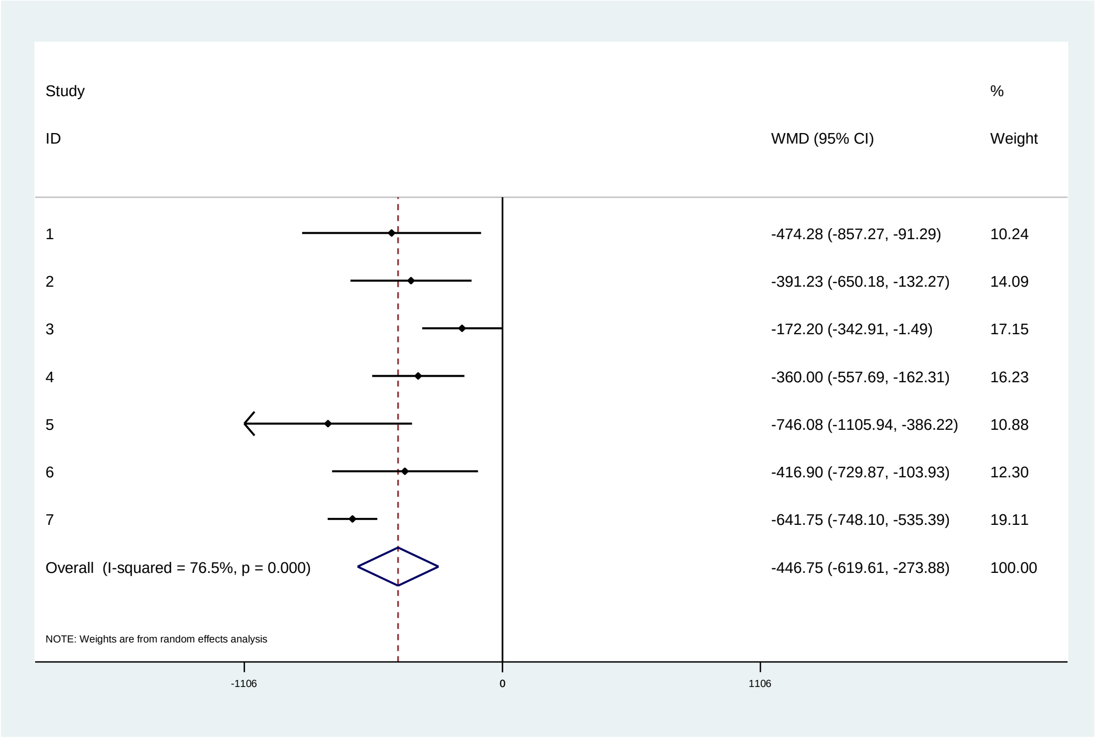

(B) Forest plot between nonsevere and severe groups for levels of B cells.

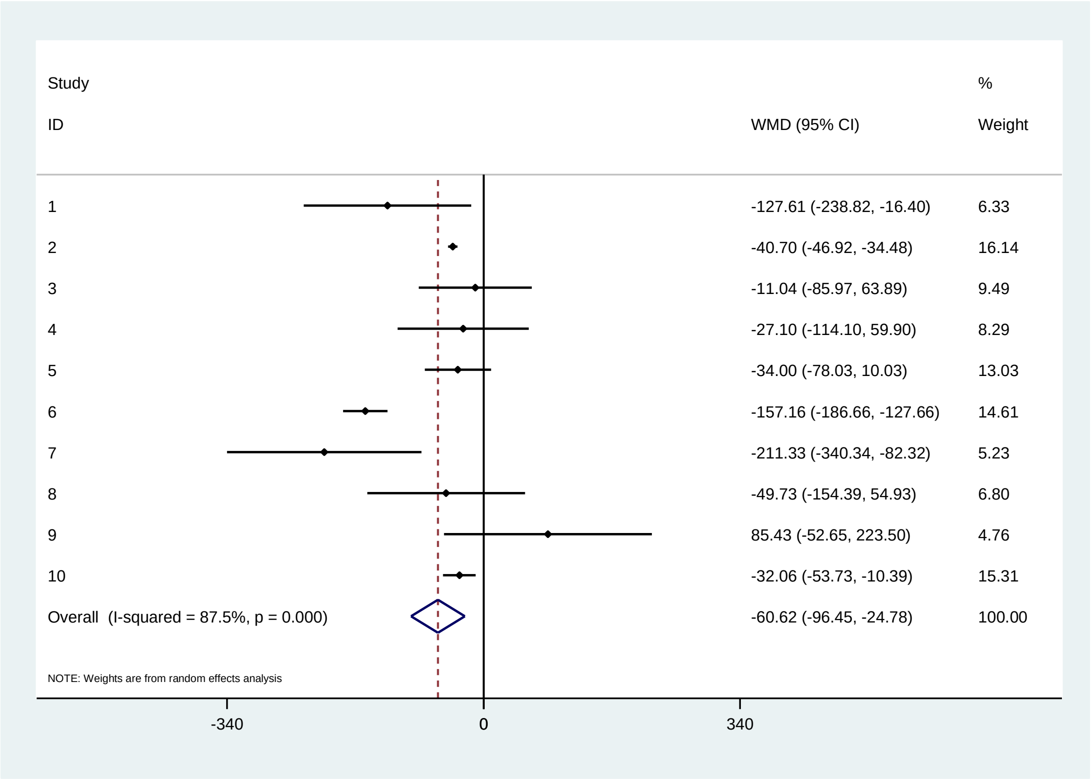

Supplement 2

(C)Forest plot between nonsevere and severe groups for levels of NK cells.

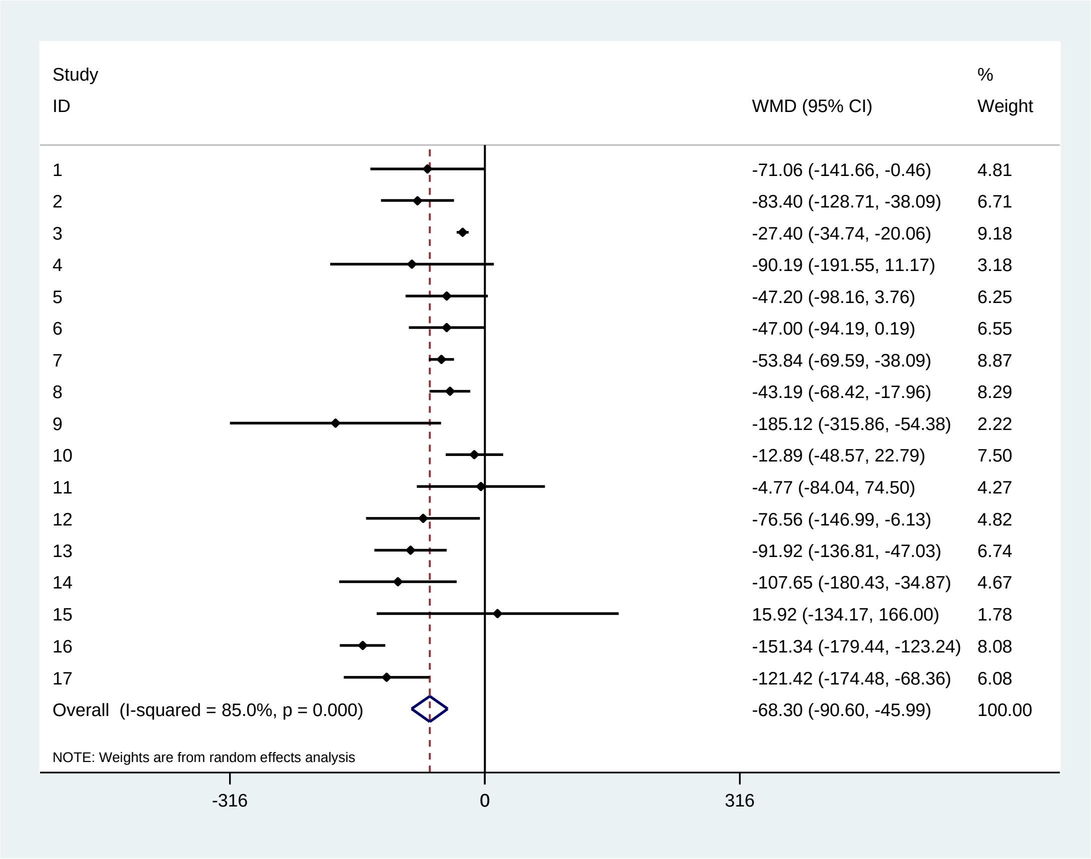

(D)Forest plot between nonsevere and severe groups for levels of CD3+ T cells.

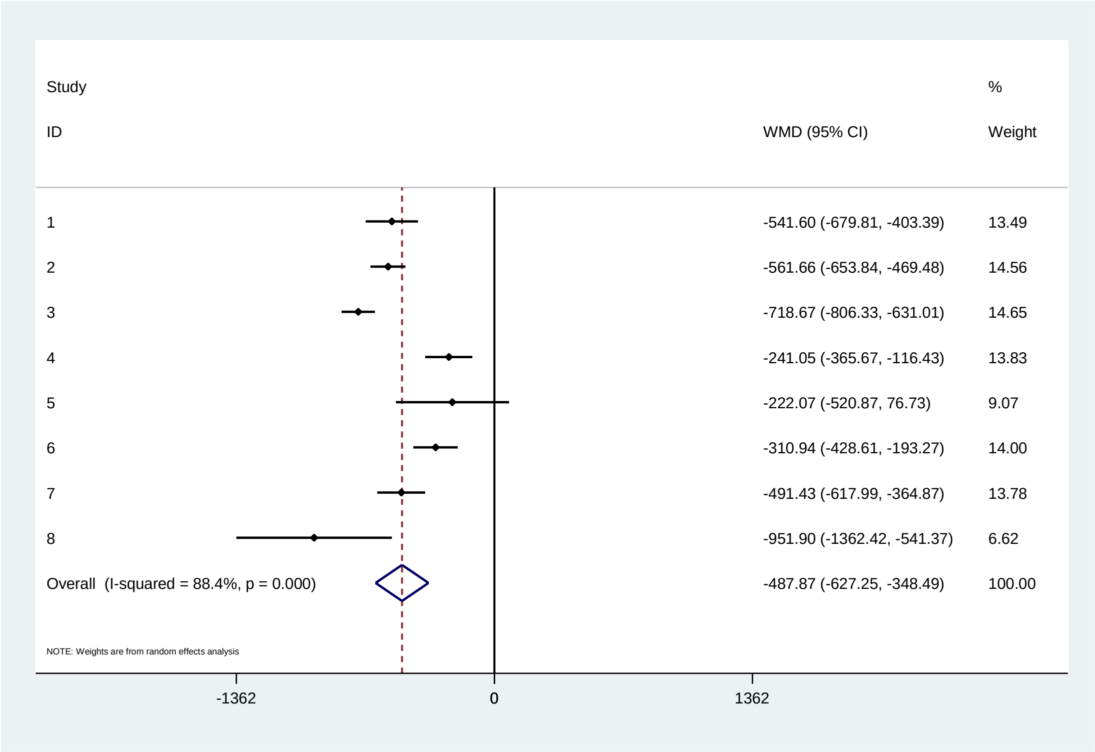

Supplement 2

(E)Forest plot between nonsevere and severe groups for levels of CD4+ T cells.

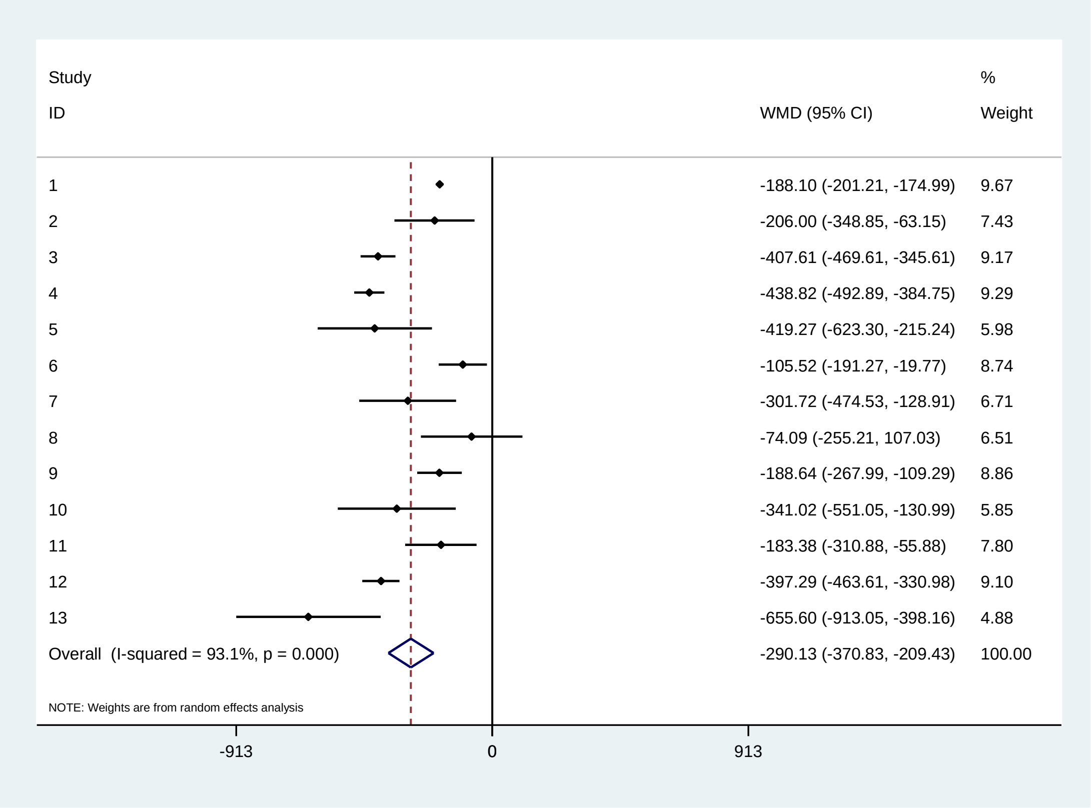

(F)Forest plot between nonsevere and severe groups for levels of CD8+ cells.

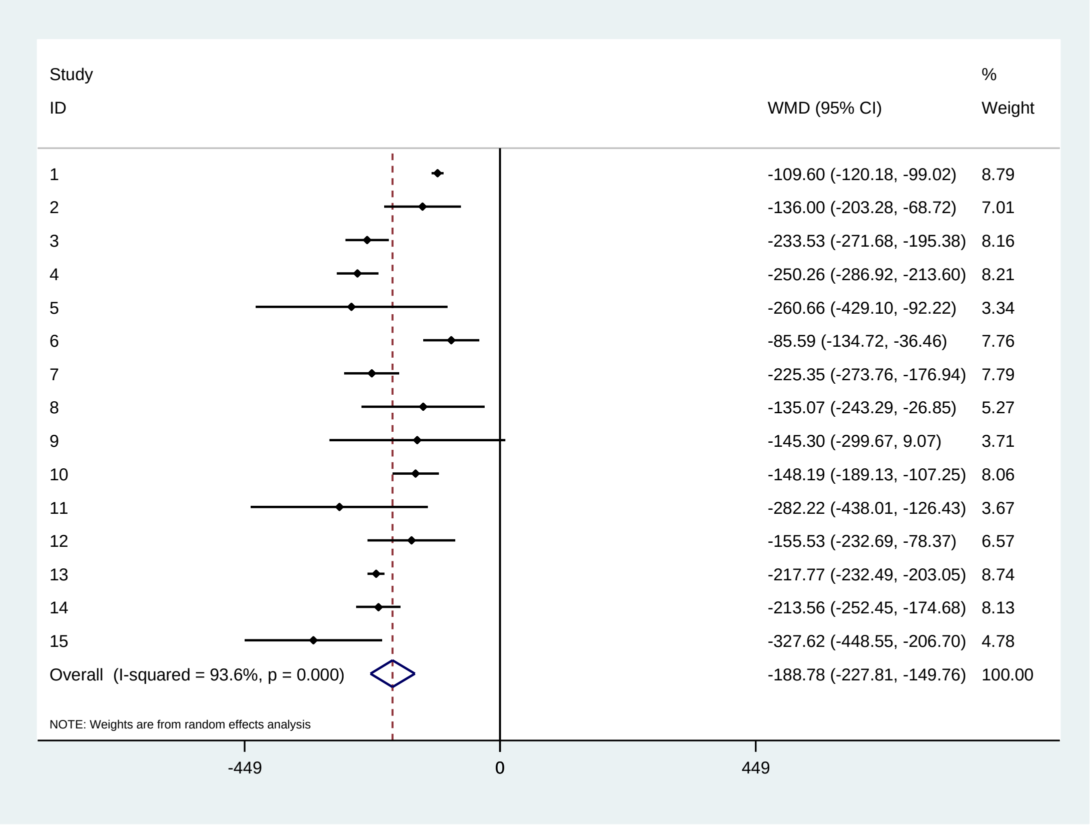

Supplement 2

(G)Sensitivity analyses between nonsevere and severe groups for levels of lymphocytes.

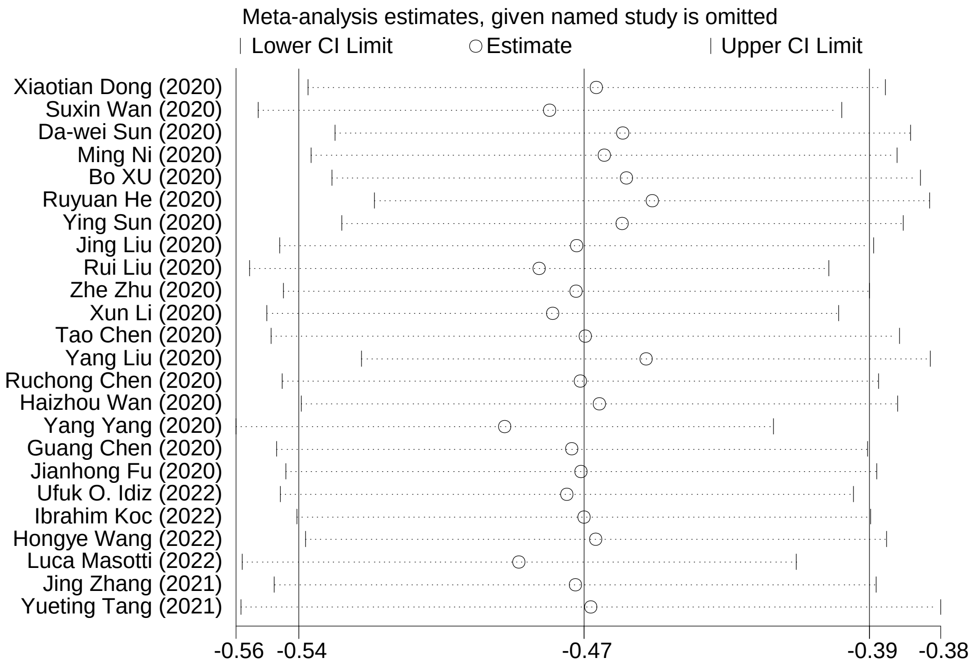

(H)Sensitivity analyses between nonsevere and severe groups for levels of T cells.

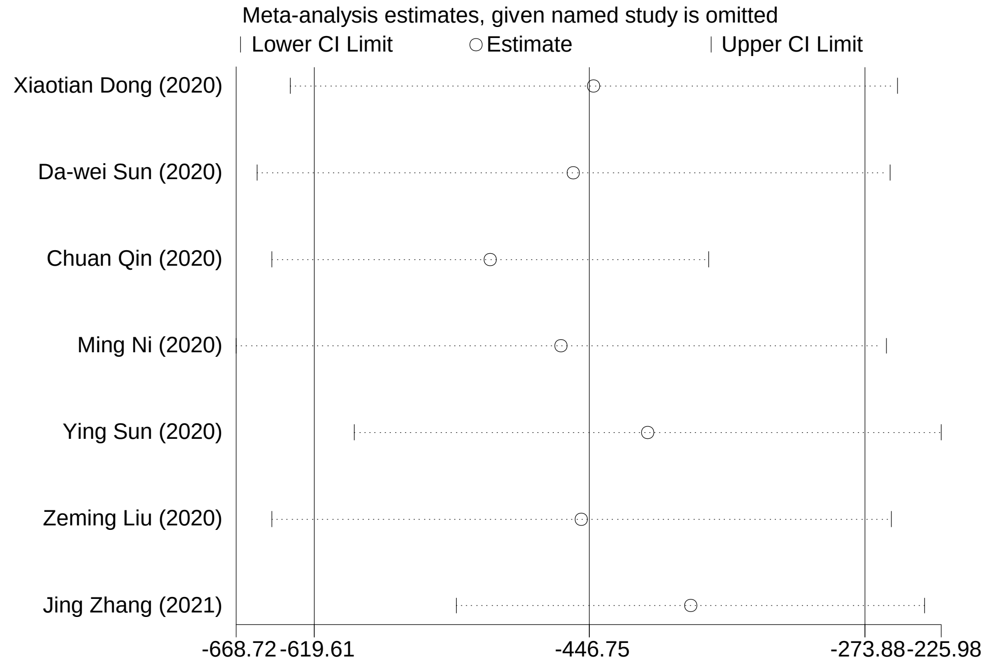

Supplement 2

(I)Sensitivity analyses between nonsevere and severe groups for levels of B cells.

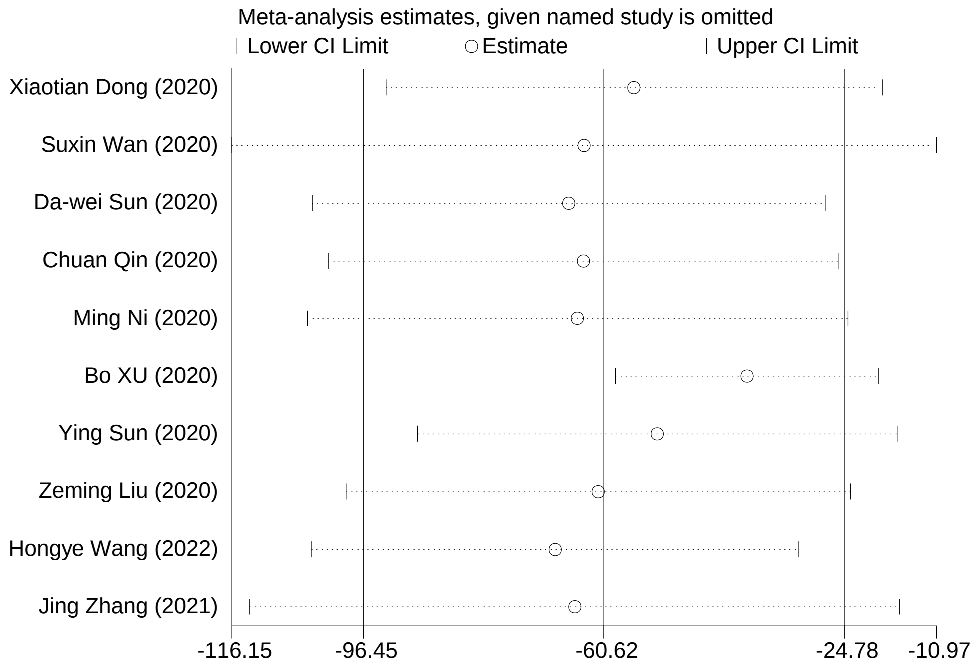

(J)Sensitivity analyses between nonsevere and severe groups for levels of NK cells.

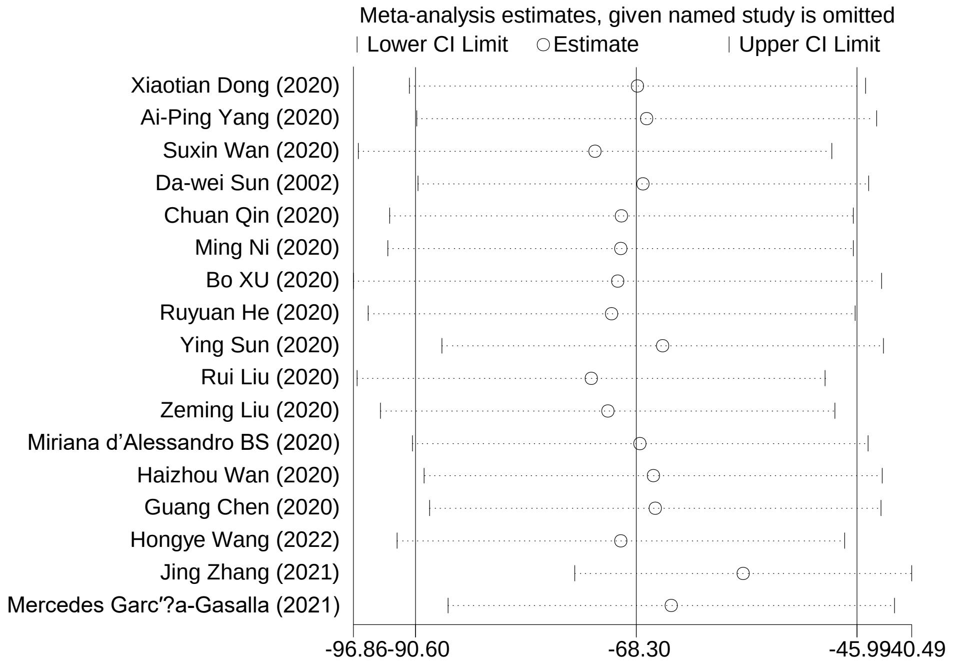

Supplement 2

(K)Sensitivity analyses between nonsevere and severe groups for levels of CD3+ T cells.

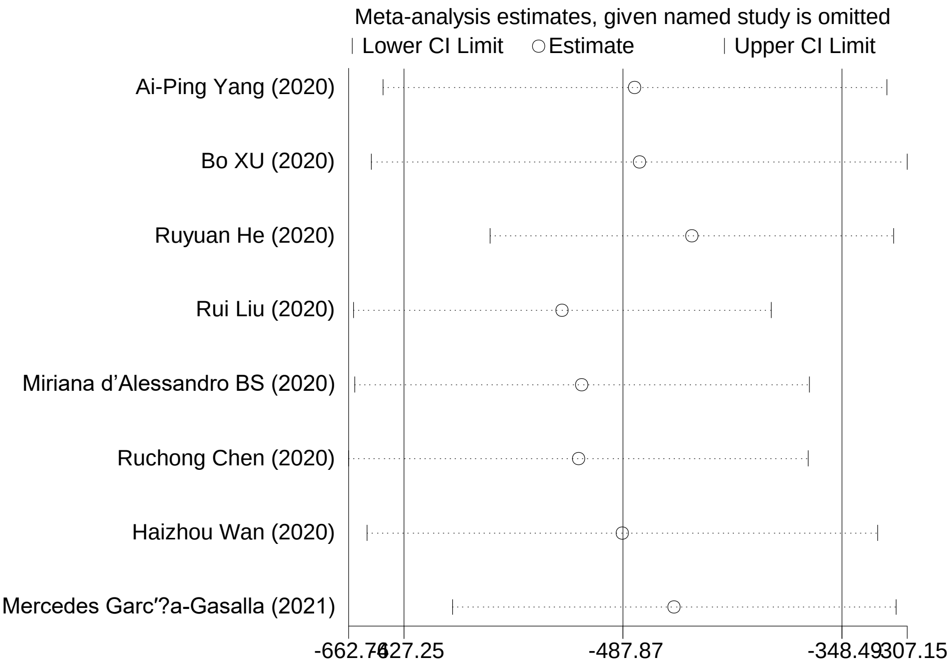

(L)Sensitivity analyses between nonsevere and severe groups for levels of CD4+ T cells.

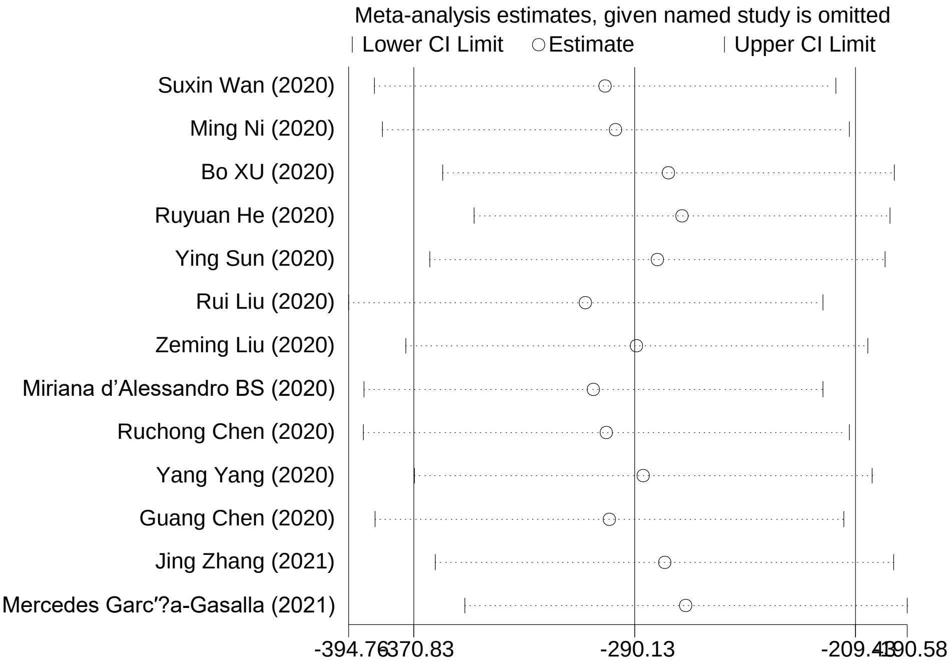

Supplement 2

(M)Sensitivity analyses between nonsevere and severe groups for levels of CD8+ T cells.

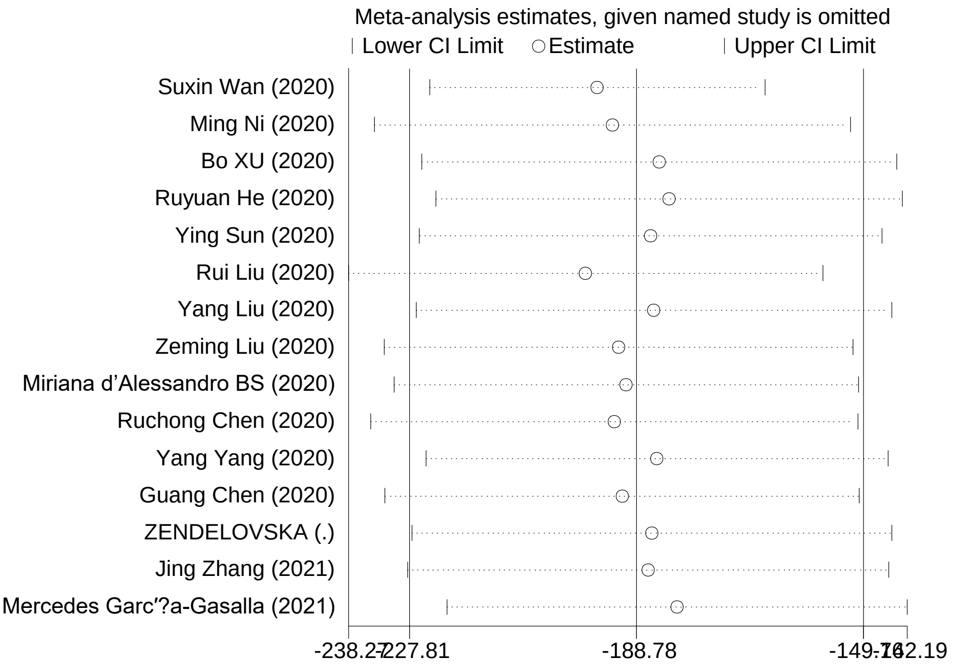

(N)Sensitivity analyses between nonsevere and severe groups for levels of neutrophil-to-lymphocyte ratio (NLR).

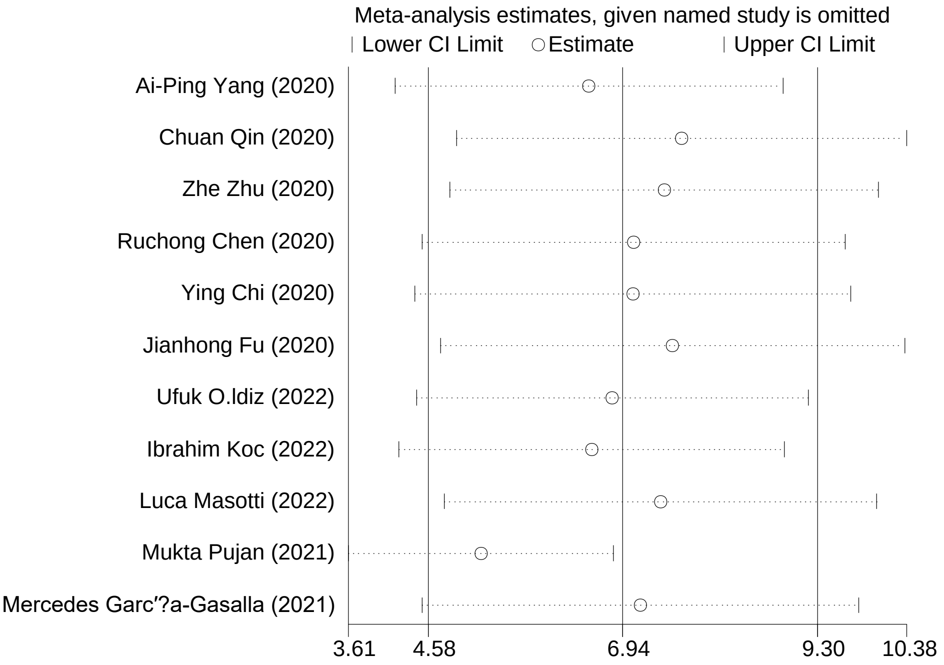

Supplement 2

(O)Publication bias by Egger' test between nonsevere and severe groups in lymphocytes.

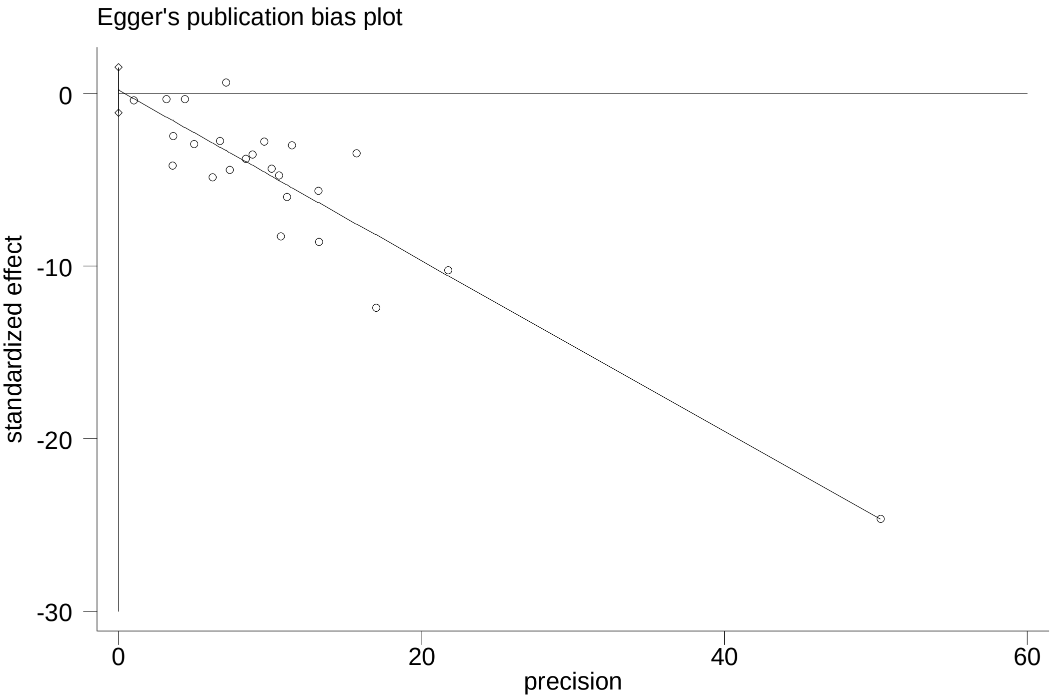

(P)Publication bias by Egger' test between nonsevere and severe groups in B cells.

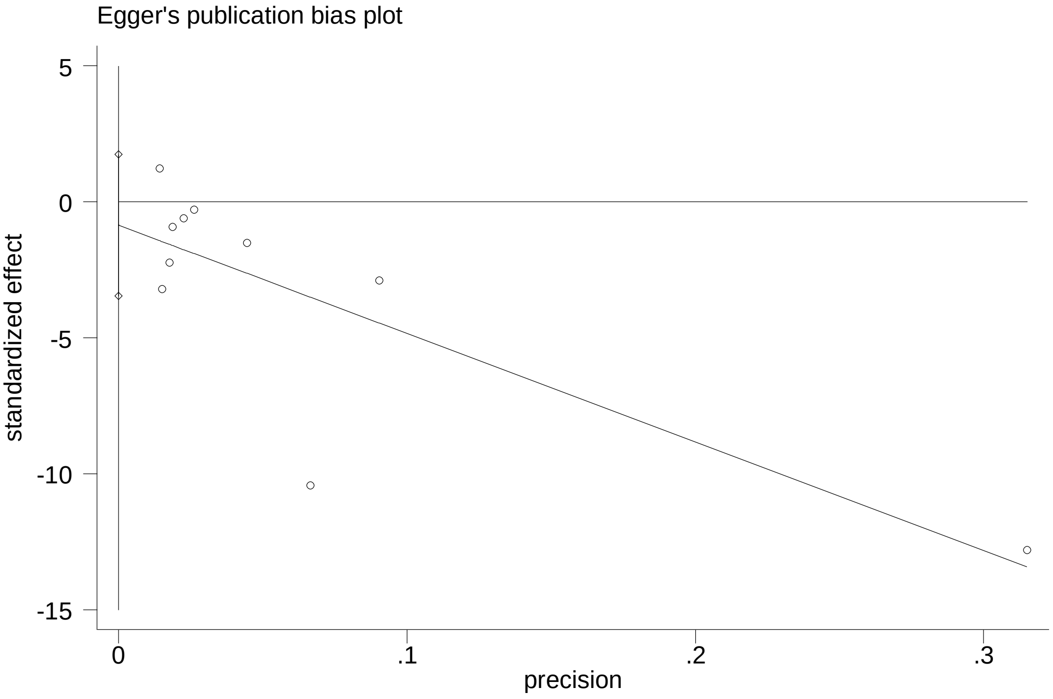

Supplement 2

(Q)Publication bias by Egger' test between nonsevere and severe groups in NK cells.

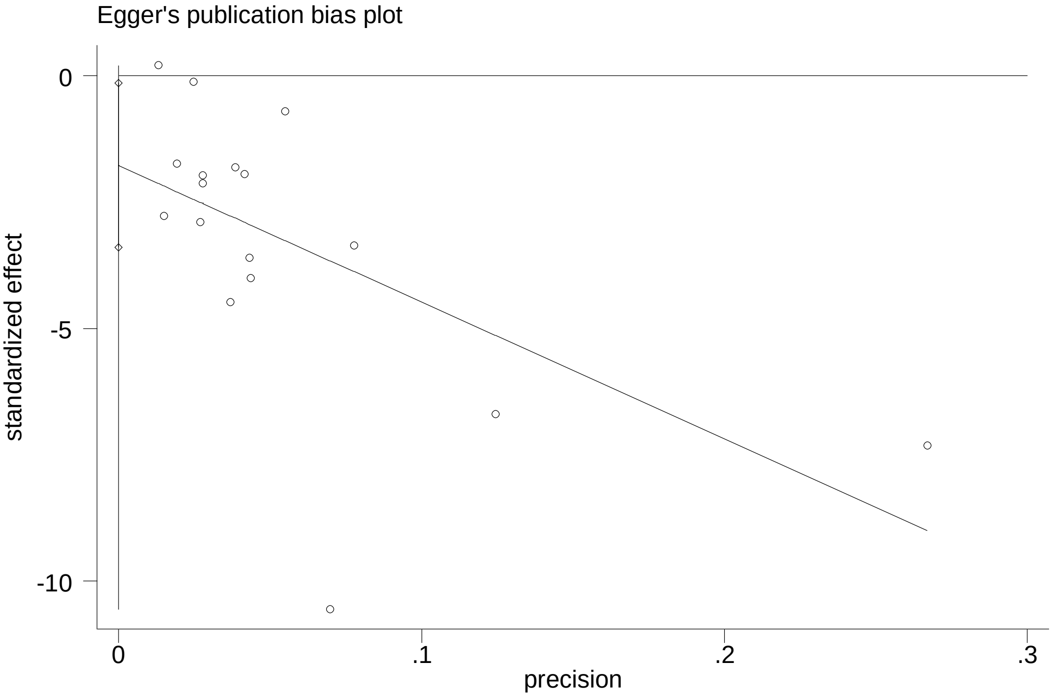

(R)Publication bias by Egger' test between nonsevere and severe groups in CD4+ T cells.

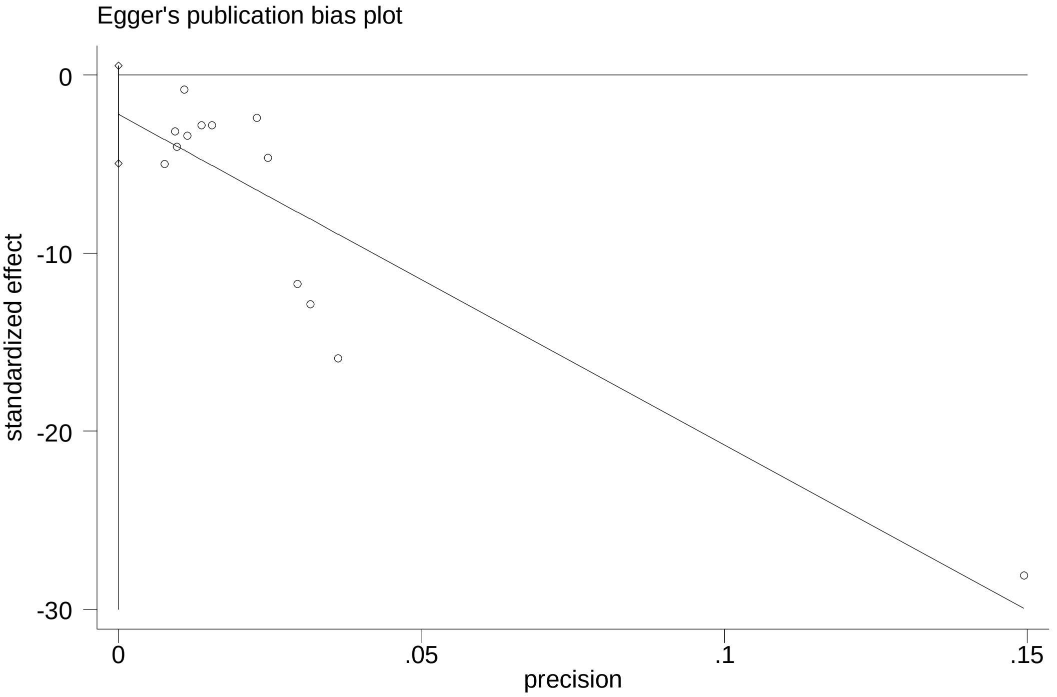

Supplement 2

(S)Publication bias by Egger' test between nonsevere and severe groups in CD8+ T cells.

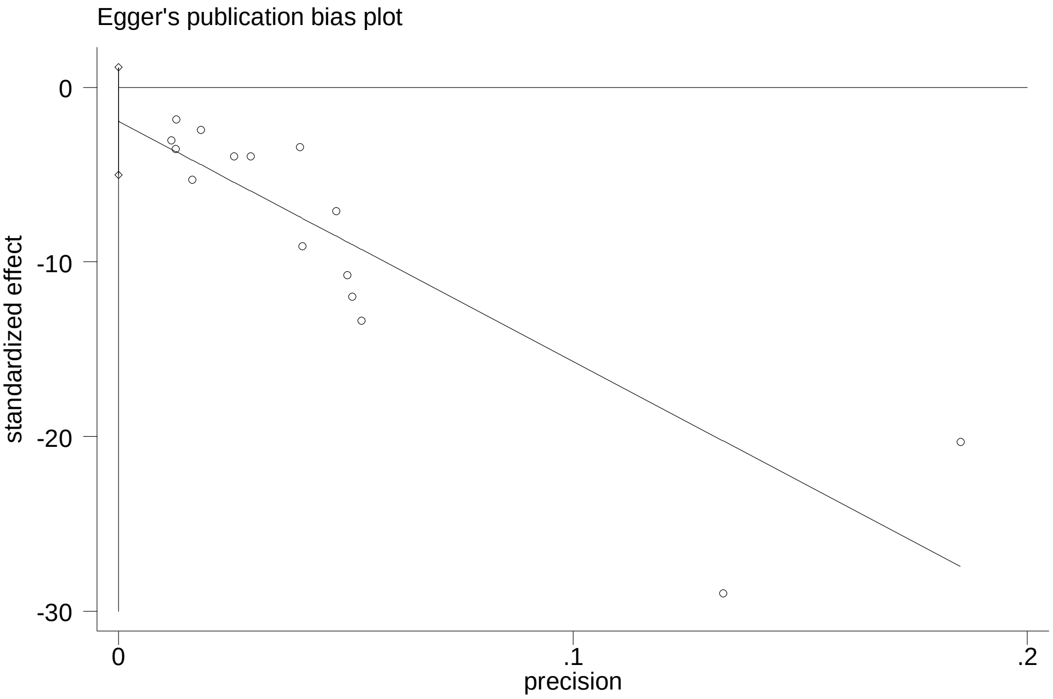

(T)Publication bias by Egger' test between nonsevere and severe groups in neutrophil-to-lymphocyte ratio (NLR).

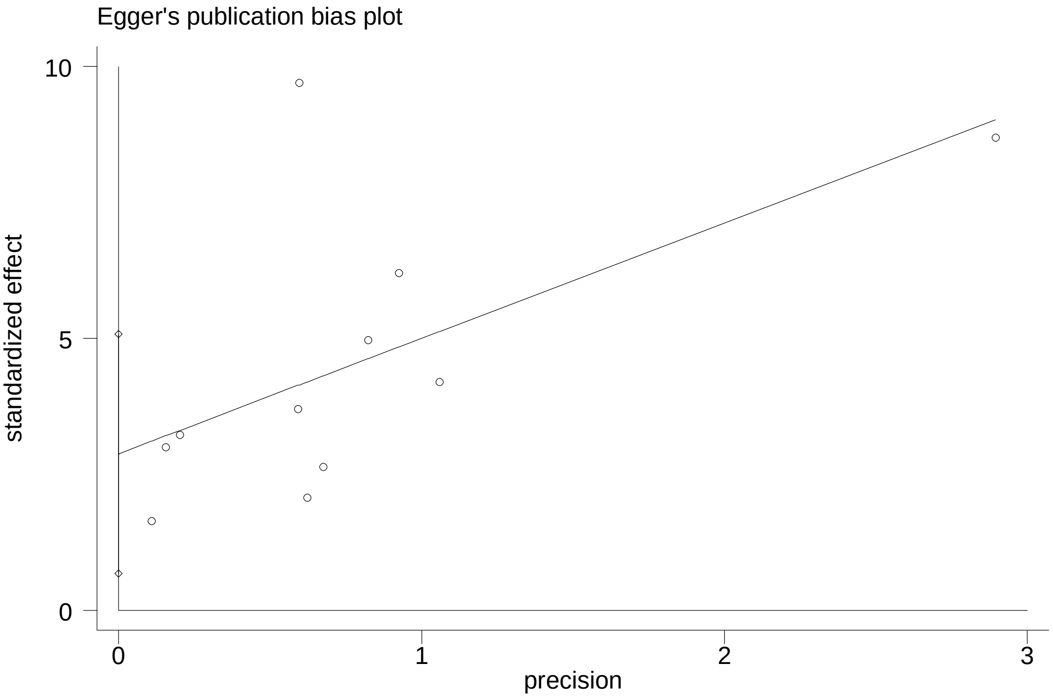

Supplement 2

(U)Funnel plot by trim-and-fill method between nonsevere and severe groups in NK cells.

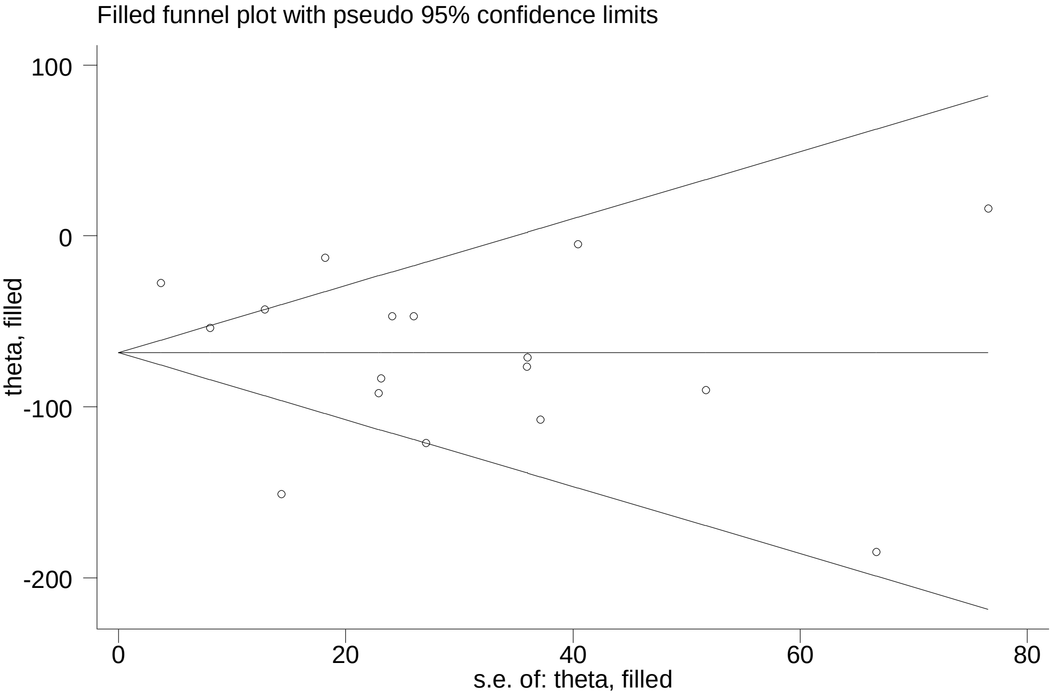

(v)Funnel plot by trim-and-fill method between nonsevere and severe groups in neutrophil-to-lymphocyte ratio (NLR).

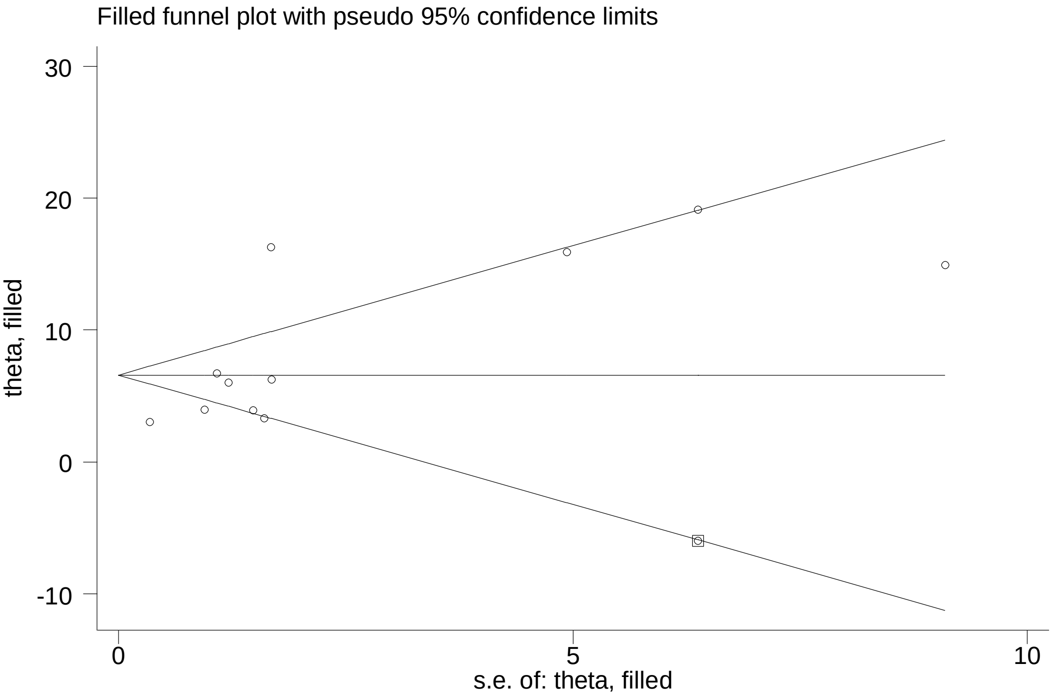

Supplement: Supplementary file 2 [file Data_Sheet_2.pdf]
