# Supplementary material for: D-dimer levels and characteristics of lymphocyte subsets, cytokine profiles in peripheral blood of patients with severe COVID-19: A systematic review and meta-analysis
Source: Front Med (Lausanne). 2022 Oct 5;9:988666. doi: 10.3389/fmed.2022.988666 (PMC9579342; doi:10.3389/fmed.2022.988666)

Supplement 3

(A)Forest plot between nonsevere and severe groups for levels of IL-2.

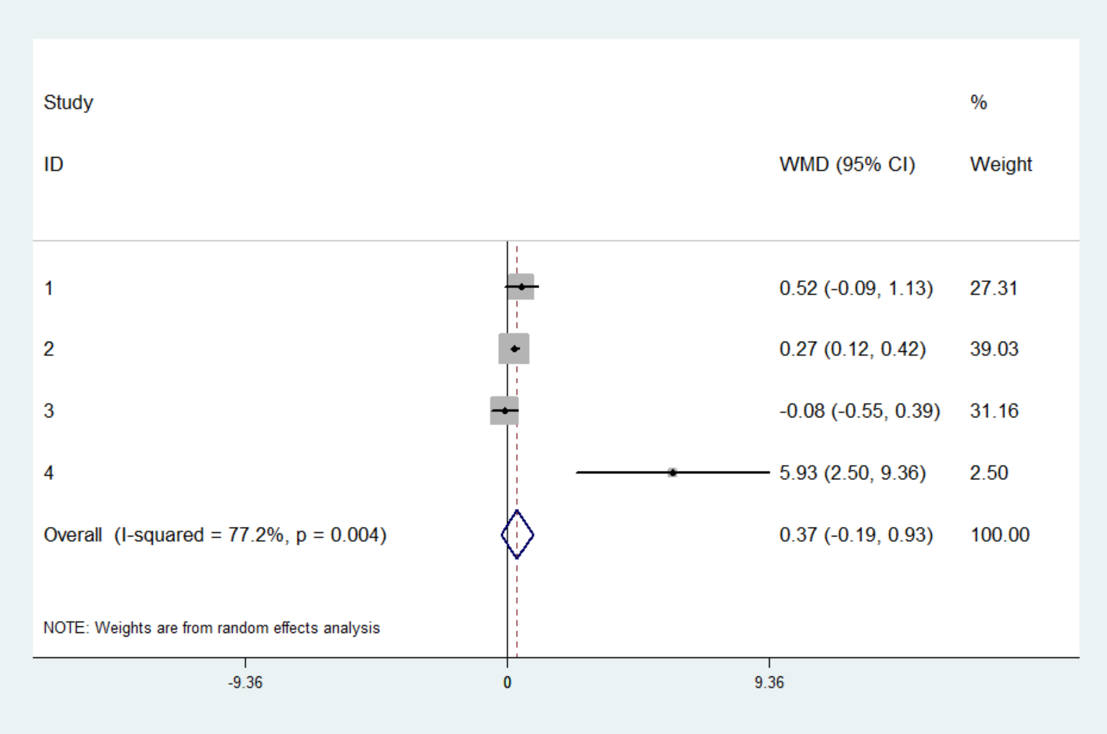

(B)Forest plot between nonsevere and severe groups for levels of IL-4.

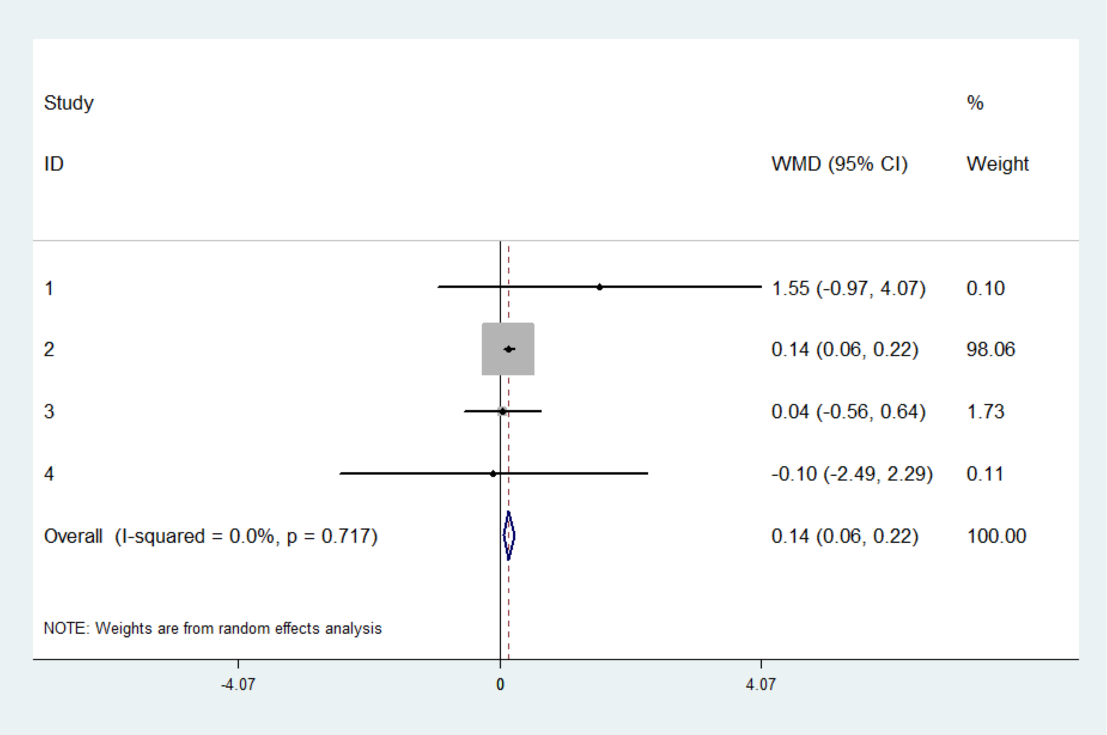

Supplement 3

(C)Forest plot between nonsevere and severe groups for levels of IL-10.

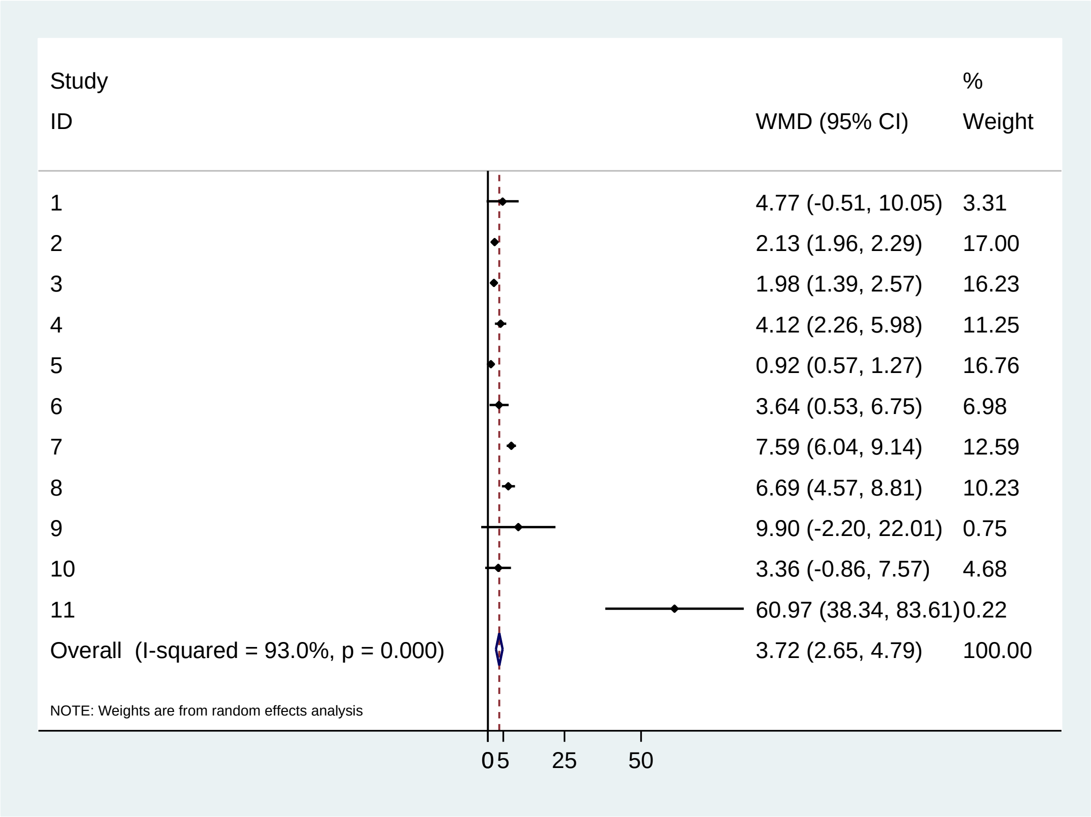

(D)Sensitivity analyses between nonsevere and severe groups for levels of IL-2.

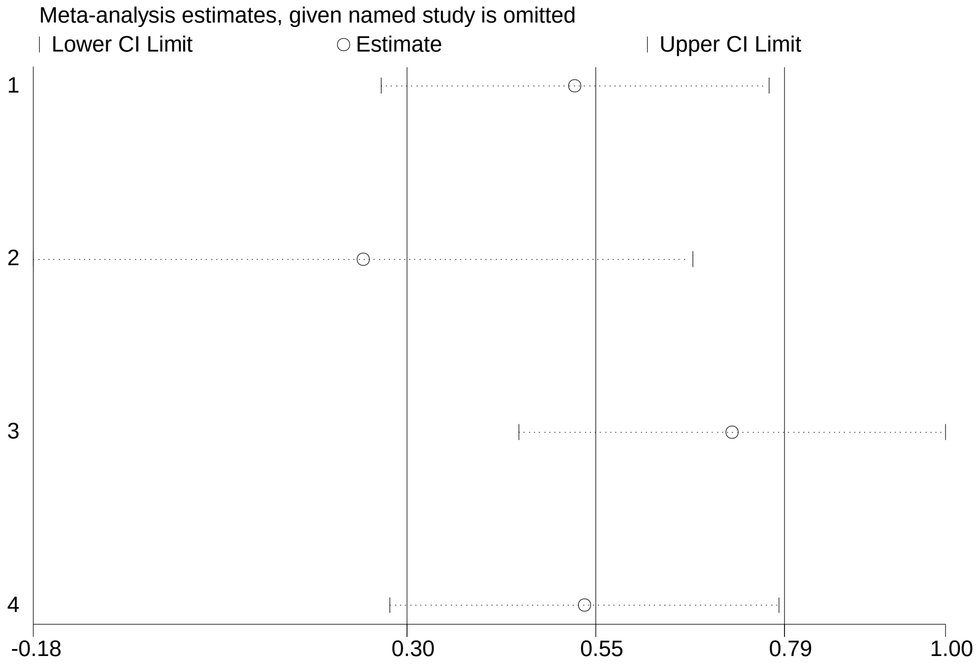

Supplement 3

(E)Sensitivity analyses between nonsevere and severe groups for levels of IL-4.

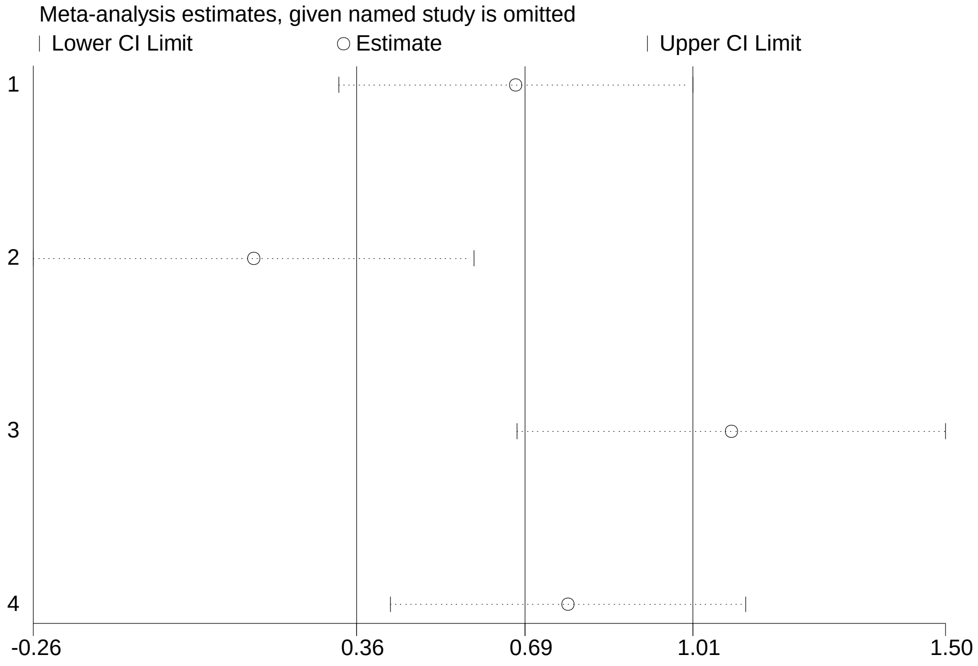

(F)Sensitivity analyses between nonsevere and severe groups for levels of IL-6.

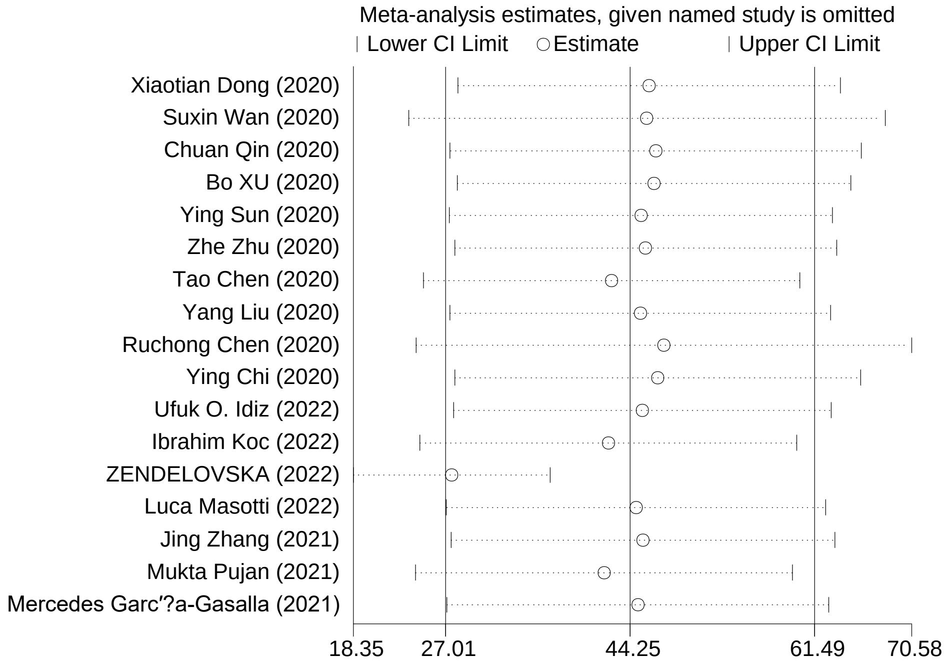

(G)Sensitivity analyses between nonsevere and severe groups for levels of IL-10.

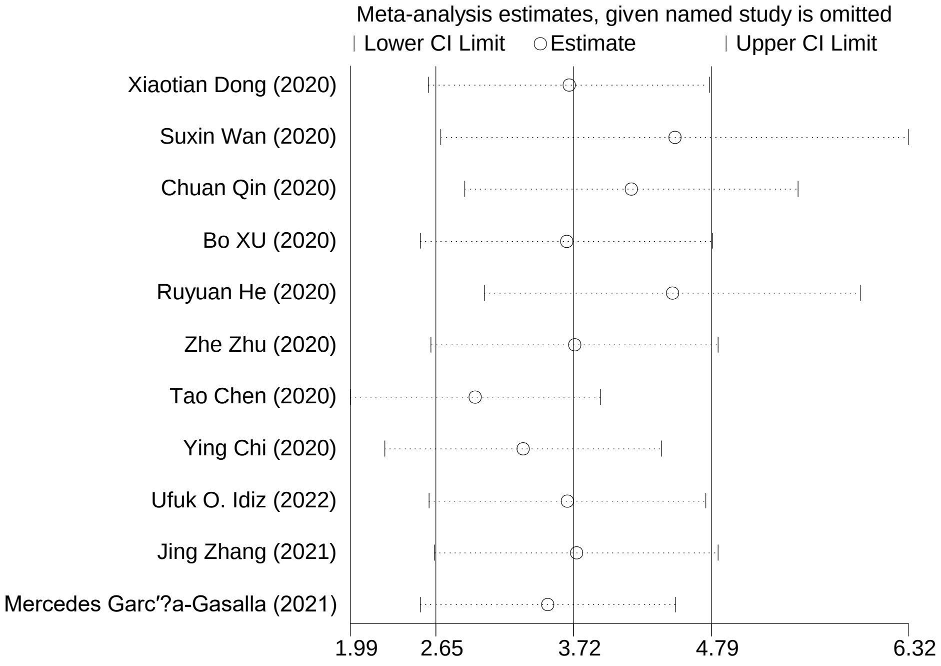

(H)Publication bias by Egger' test between nonsevere and severe groups in IL-6.

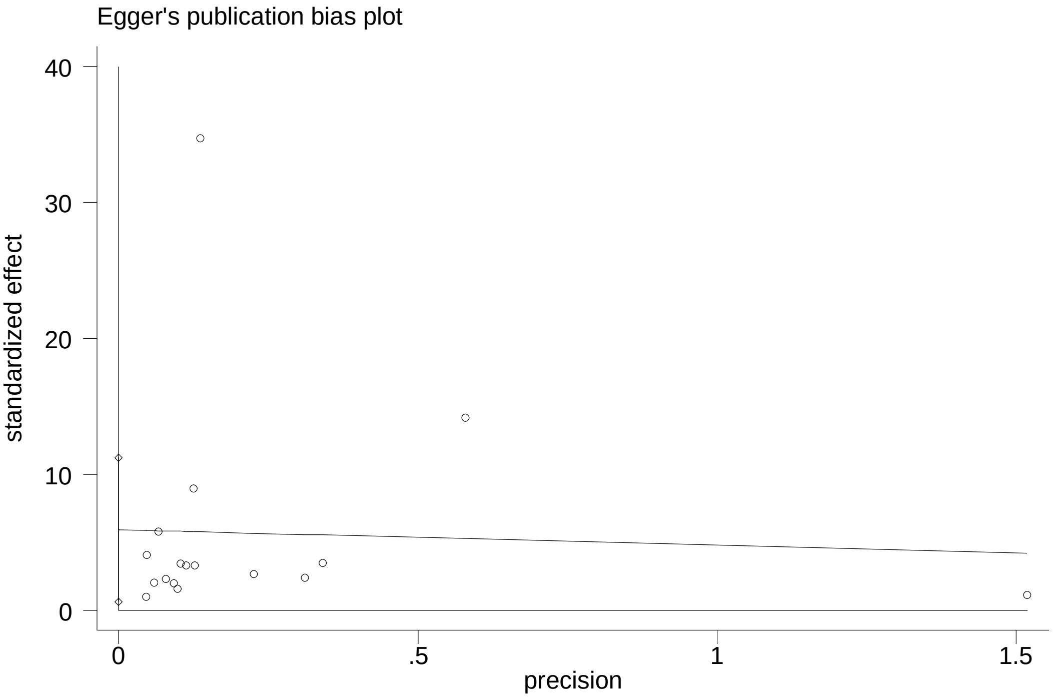

Supplement 3

(I)Publication bias by Egger' test between nonsevere and severe groups in IL-10.

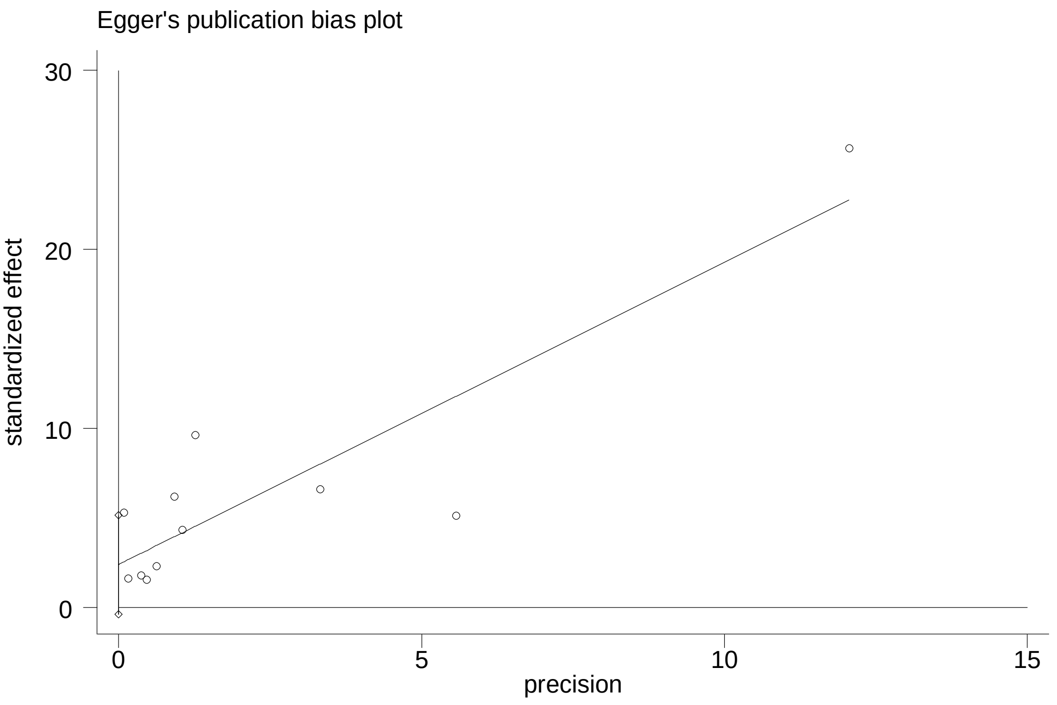

(J)Funnel plot by trim-and-fill method between nonsevere and severe groups in IL-6.

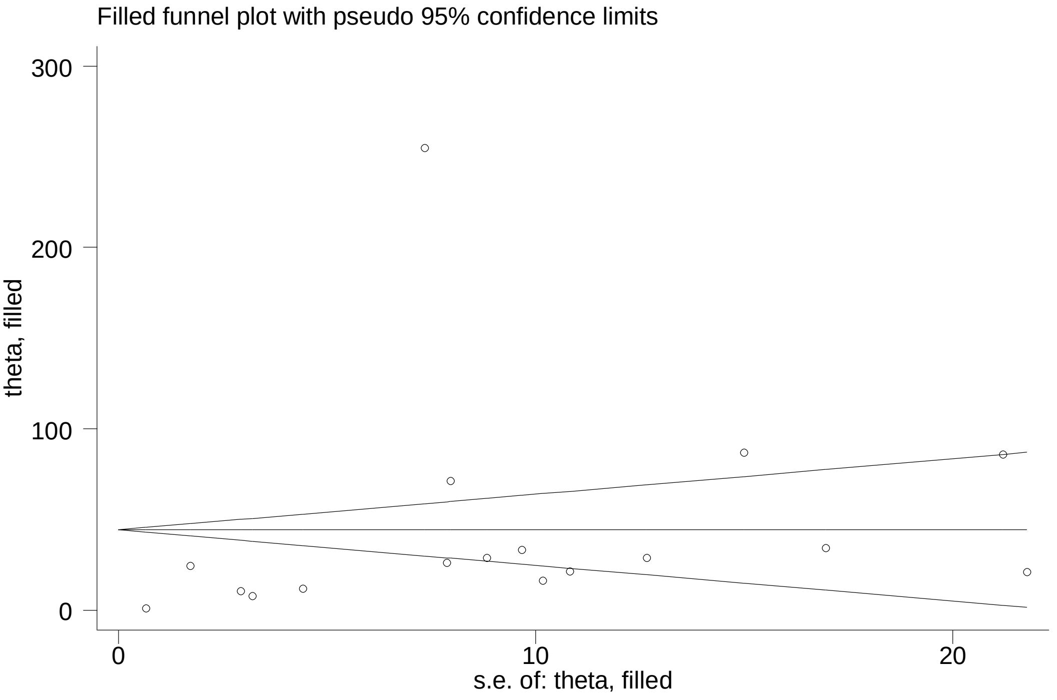

Supplement: Supplementary file 3 [file Data_Sheet_3.pdf]
